# Novel 11-Substituted Ellipticines as Potent Anticancer Agents with Divergent Activity against Cancer Cells

**DOI:** 10.3390/ph12020090

**Published:** 2019-06-14

**Authors:** Charlotte M. Miller, Elaine C. O’Sullivan, Florence O. McCarthy

**Affiliations:** School of Chemistry, Analytical and Biological Chemistry Research Facility, University College Cork, Western Road, T12 K8AF Cork, Ireland; charlotte.herstad@dynea.com (C.M.M.); elosullivan@hotmail.com (E.C.O.)

**Keywords:** ellipticine, anticancer, heterocyclic chemistry, indole, NCI screen, topoisomerase II

## Abstract

Ellipticines have well documented anticancer activity, in particular with substitution at the 1-, 2-, 6- and 9-positions. However, due to limitations in synthesis and coherent screening methodology the full SAR profile of this anticancer class has not yet been achieved. In order to address this shortfall, we have set out to explore the anticancer activity of this potent natural product by substitution. We currently describe the synthesis of novel 11-substituted ellipticines with two specific derivatives showing potency and diverging cellular growth effects.

## 1. Introduction

Cancer is a collection of diseases defined by the proliferation of cells in an unregulated and inappropriate manner leading to tumours and eventual mortality. Within this broad definition it is evident that the control of cell growth is a key mechanism as cell growth can be restricted, proliferation can be stopped and cells can be killed by multiple anticancer agents, one of which (ellipticine) is explored further here [1].

**Figure 1:**
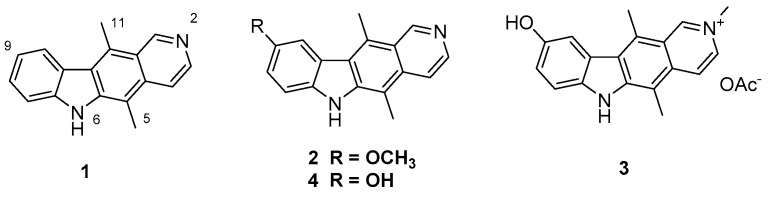
Structures of Ellipticine **1** and related anticancer agents.

Ellipticine **1** (5,11-dimethyl-6*H*-pyrido [4,3-*b*]carbazole, Figure 1) is a natural product which was isolated in 1959 from a small tropical evergreen tree (*Ochrosia elliptica*) by Goodwin et al. [2]. The extract also contained a number of other alkaloids, including the related 9-methoxyellipticine **2**. Since its isolation, the planar tetracyclic structure of ellipticine has been the focus of extensive chemical and pharmacological research [3]. Over the last 60 years, ellipticine and its derivatives have been identified with potent anticancer activity and been subject to clinical trials. Celiptium (9-hydroxy-*N*-methylellipticinium acetate) **3** and 9-hydroxyellipticine **4** both progressed to phase II clinical trials though these were subsequently discontinued due to side effects and efficacy [4,5,6,7]. 

The mechanisms by which ellipticine exerts its anticancer activity are particularly diverse. These include topoisomerase II inhibition with associated DNA intercalation, well established in the literature and clinically relevant [8,9,10]. Cytotoxic ellipticine metabolites via biooxidation at a number of positions on the tetracycle and subsequent adduct formation have also been identified as responsible for some of the effects [11]. Recently, ellipticines have been described to interact with kinases such as c-Kit, AKT and CK2, and to influence the p53 tumour suppressor, all of which have key roles in cell growth and progression [12,13,14]. Ellipticines have also been discovered to disrupt RNA polymerase I transcription and stabilize pharmaceutically relevant G-quadruplexes and so there is significant and continued interest in the biological effects of this compound class [15,16]. 

**Figure 2:**
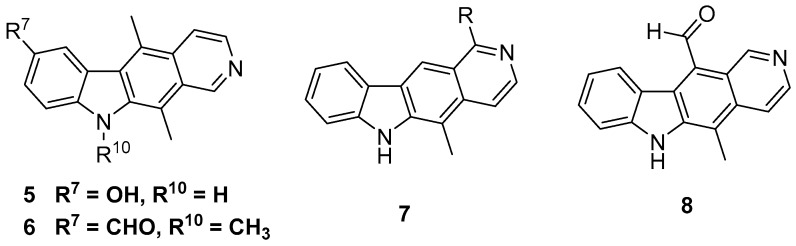
Substituted isoellipticines (**5** and **6**) with in vivo activity, Olivacine **7** and 11-formylellipticine **8**.

It is therefore evident that ellipticine and its derivatives exhibit multimodal activity with simple substitutions on this template eliciting diverse effects. Despite their potency, there has been little exploration of the functional space in this natural product outside of the 1-, 2-, 6- and 9-positions in order to answer fundamental questions in pharmacology and effect on cells. A programme of research has been initiated in this group in order to evaluate the effect of novel substitution patterns on the ellipticine framework. This has led to the recent discoveries of substituted ellipticine and isoellipticines as new investigational probes with diverse pharmacology and exceptional in vivo activity (**5** and **6**, Figure 2) [17,18,19,20].

The 11-position of ellipticine has received little attention from medicinal chemists despite evidence that it may be a key position in order to affect the bioactivity and crucially selectivity of pharmacology [21]. The activity of the closely related olivicine series of compounds signifies that removal of the methyl group does little to affect the potency of the template especially in regard to topoisomerase activity and DNA intercalation (**7**, R = CH_3_, Figure 2) [22,23]. An olivacine derivative, S16020 (**7**, R = carboxamide) has also progressed to phase I clinical trials before being discontinued [24]. Despite synthesis of 11-formyl ellipticine **8** (Figure 2) almost 30 years ago, no concerted effort to functionalise this position is recorded in literature other than a carboxamide and a methyl ketone (only four reported compounds in total with carbonyl at 11-position). 

Molecular modelling of ellipticine in the active site of biological targets has postulated that 11-substitution with bulky or mobile functional groups would inhibit the intercalation process and may reduce a number of undesirable effects associated with treatment [25]. This position has also been implicated in the process of DNA adduct formation via oxidation [11].

To this end, we set out to functionalise the parent heterocycle and to exploit its known anticancer activity: it has obvious potency across numerous spheres of biology and affects cells from development to apoptosis [4]. Substitution of 11-position is an important step towards full SAR profiling of the cellular effects of ellipticine derivatives. We document here the initial scoping of this chemical space and probe of the anticancer activity through known ellipticine mechanisms.

## 2. Results and Discussion

### 2.1. Synthesis of 11-Substituted Ellipticine Derivatives

Since its isolation in 1959, the total synthesis of ellipticine has been accomplished many times and most recent work in the area has focused on preparation of analogues and derivatives of ellipticine [3]. At the outset of this work there existed relatively few examples of 11-substituted ellipticines. Gribble et al. had published a very versatile and efficient route in 1989, giving access to 5- and 11-substituted ellipticines [26,27,28,29]. This was further elaborated by Modi et al. to give 11-formylellipticine 8 and this compound was chosen as the starting point for the current synthetic work [30,31]. 

#### 2.1.1. Modification of 11-formylellipticine

**Scheme 1:**
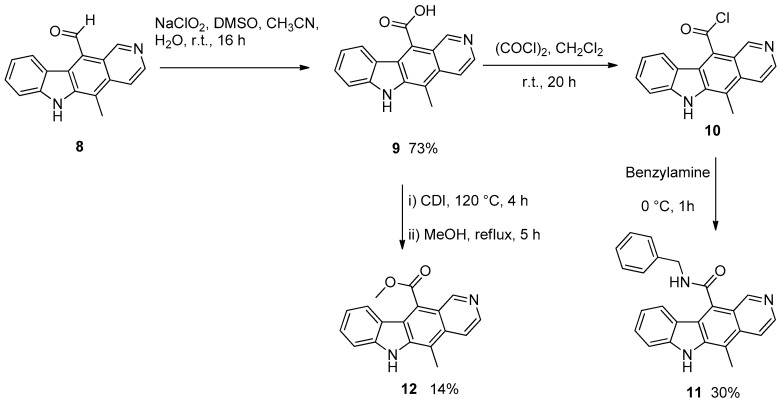
Formation of 11-substituted ellipticine amide **11** and ester **12**.

11-Formylellipticine 8 was synthesised as previously described and initially the focus was to provide a carboxylic acid handle for modification (Scheme 1) [30,31]. Hence, 8 was subjected to oxidation under a number of conditions with the most successful conversion to carboxylic acid 9 achieved using sodium chlorite and dimethylsulfoxide (73% yield). Subsequent conversion to acid chloride 10 allowed for introduction of amide functionality which was a key target in this series and the benzylamine derivative 11 was prepared in two steps in 30% yield. The methyl ester 12 was also prepared via similar methodology using carbonyldiimidazole.

**Scheme 2:**
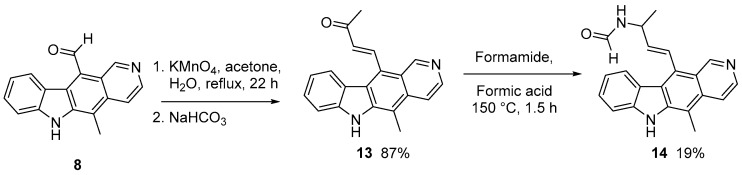
α,β-Unsaturated ketone condensation product **13** from acetone solvent.

Interestingly, while attempting to form the acid initially with other oxidants the expected product was not isolated. On treatment of 8 with potassium permanganate under standard conditions the reaction resulted in base condensation of acetone and isolation of the novel α,β-unsaturated ketone 13 in 87% yield (Scheme 2). 

Attempts at cyclisation of compound 13 using aluminium chloride in dichloromethane or chloroform to form a new ring system were unsuccessful and unreacted starting material was recovered. However, a Leuckart reaction gave the formamide 14 in 19% yield, the low yield owing to difficulties in purification. However, the unsaturated ketone 13 provides an interesting template for potential covalent adduct formation and will be revisited in the future. 

#### 2.1.2. Modification of the 9-position of 11-substituted Ellipticines

**Scheme 3:**
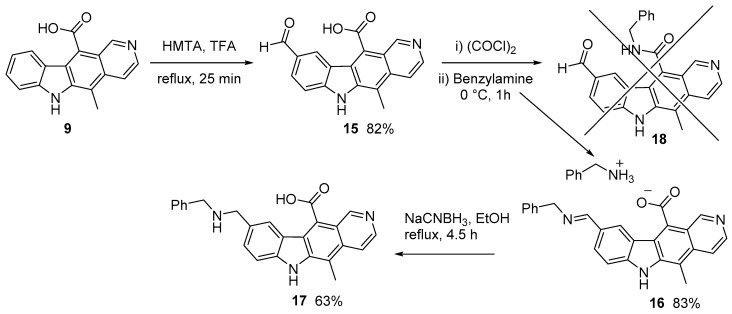
Synthesis of 9- and 11-substituted ellipticines.

Given the extensive literature background in 9-substituted ellipticines and the recent developments in this area we next turned our focus to functionalization of this position. Initially, carboxylic acid 9 was derivatised via a Duff reaction to give the aldehyde 15 in 82% yield (Scheme 3) [32]. 

Subsequent reaction of 15 with nucleophiles opens up opportunities for potential synthesis at either the 9- or the 11-position (Scheme 3). To probe the 11-position further, acid chloride formation prior to the addition of benzylamine (as seen in Scheme 1) was undertaken with the aim of generating amide 18. In this reaction, the product isolated was the imine 16 (83% yield) which was identified after full characterization. Confirmation of structure was given by subsequent treatment with aqueous acid which hydrolysed the product to starting material 15. 

Additional conformation of the imine 16 was provided by reduction of the imine with sodium cyanoborohydride in ethanol to give the novel amine 17. 

2.1.3. 1H NMR Analysis of 11-Substituted Ellipticines

A summary of the ^1^H NMR analysis is provided in Supplementary Materials (Figures S1–S3). Of note is the influence of conversion from aldehyde to carboxylic acid at the 11-position with corresponding upfield shift of C1 and C10 protons (reversed on formylation of the 9-position). The shift of the C10 proton is again evident on conversion of carboxylic acid to amide and ester.

### 2.2. Biological Evaluation of 11-Substituted Ellipticines

Given the interest in new ellipticines, several of the novel 9- and 11-substitued ellipticines were evaluated for their biological activity in cellular screens and by gel electrophoresis. In the first instance, inhibition of topoisomerase II (a well-known target of ellipticines) was assessed. 

#### 2.2.1. Inhibition of Topoisomerase II

**Figure 3:**
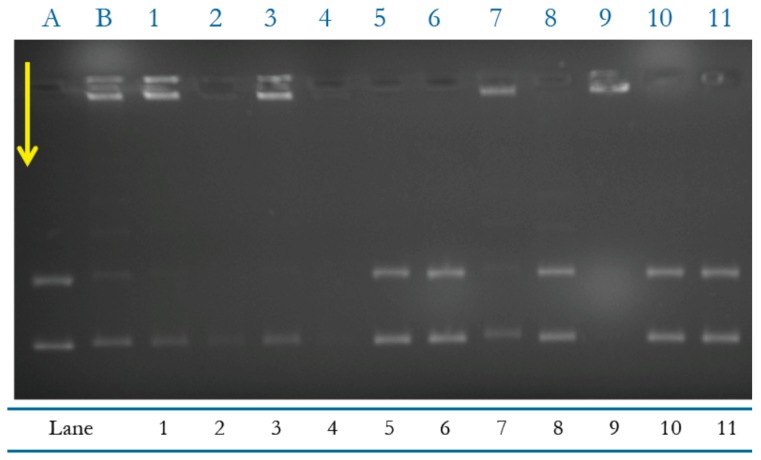
Screen of topoisomerase II inhibitory activity of the ellipticine derivatives at 100 µM. A = Positive Control (kDNA + ATP + Topo II); B = Negative Control (kDNA + ATP + Topo II + 100 µM Ellipticine); Consequent lanes all screened at 100 µM: 1 = Ellipticine 1; 2 = 9-Methoxyellipticine 2; 3 = 9-Formylellipticine 19; 4 = 9-Hydroxyellipticine 4; 5 = 8; 6 = 9; 7 = 13; 8 = 15; 9 = 16; 10 = 17; 11 = 11.

Topoisomerase II inhibition is a clinically used cancer treatment and is considered to be a key mechanism of action for ellipticines [9]. Topoisomerase II has key functions in the change of topological structure of DNA and hence cell replication which can be evaluated using a decatenation assay. As expected, the planar ellipticine 1 and the simple 9-substituted ellipticines (2,4,19) all displayed excellent inhibition of topoisomerase II at 100 µM (Figure 3). On assessment of the 11-substituted ellipticines, the majority were inactive against topoisomerase II but compounds 13 and 16 showed the most promise. α,β-Unsaturated ketone 13 and 9-substituted imine 16 both represent new templates for discovery. Both compounds were evaluated in a subsequent three-fold dilution assay (at 100, 10 and 1µM) where it became apparent that activity reduced significantly below the initial test concentration. Notwithstanding this, the emergence of two diverse templates for topoisomerase inhibition will be of keen interest to the field and future efforts will aim to increase the potency of this interaction. 

#### 2.2.2. Inhibition of Cancer Cell Growth

Having identified some limited topoisomerase activity, screening of the 11-substituted ellipticine test set was next conducted by assessment of inhibition of cancer cell growth. Initial evaluation of anticancer activity at the National Cancer Institute focuses on the effect of each individual compound on the growth of up to 60 cancer cell lines at a specific concentration (10 µM). The results are summarised in Table 1 (see Supplementary Material for full data, Figures S5–S10). 

##### Inhibition of Cancer Cell Growth—One Dose Assay

National Cancer Institute (NCI) evaluation of 11-substituted ellipticines identifies significant effects on the growth of the 60-cell line panel with mean growth values ranging from 18% to 106%. The Mean Growth percent is a reference tool whereby screening at 10 µM concentration is used to filter active anticancer compounds. Of the six compounds tested, two (11-substituted amide 11 and conjugated ketone 13) achieved Mean Growth percentages of <25% and fulfilled the requirements for progression to the five dose assay (Table 1). Of the other four compounds growth percentages were between 96% and 106% and hence have no effect on the growth as an average across the 60 cell lines. Compounds 9, 15, 16 and 17 elicited very similar effects being most active against renal cancer cell line UO-31 (range 63%–76% growth) and breast cancer cell lines MCF7 and MDA-MB-231/ATCC (range 68%–87% growth) but have little effect on the other cell lines of the screen (Figures S5, S8–S10). 

In contrast, benzylamide 11 and ketone 13 had significant anticancer effects with differing cellular profiles. Ketone 13 is associated with a non-specific pattern with the range of cell growths (lowest to highest) of 77% whereas benzylamide 11 has a range of 165% and there is an evident difference in cell line effects. At 10 µM, compound 11 is cytotoxic (negative growth percentage) to 15 of the 60 cell lines whereas compound 13 to only four cell lines. It is noticeable that a large number of cell lines are refractory to benzylamide 11 which suggests there may be a targeted effect. It is also obvious from this preliminary screen that there is chemical space at the 11-position of ellipticine to accommodate anticancer activity.

##### Inhibition of Cancer Cell Growth—Five Dose Assay

The progression to evaluation at five dose assay confirmed the potency of both 11-substituted ellipticines 11 and 13 and their specific effects on cells seen in the one dose screen. Benzylamide 11 exerts a broad range of activity from cytostatic to cytotoxic at dose ranges from 1 to 100 µM (Table 2; Figure S11). In specific, the activity of this compound against HOP62, SNB75, OVCAR-3, OVCAR-4 and 786-0 is exceptional with more than 50% cytotoxicity measured after 48 h incubation (at 10 µM). Growth is restricted across the other cell lines but there is clear selective cytotoxicity. In addition it can be seen (Figure S12) that a number of cell lines are refractory with no evident effect on cell growth of cancer subtypes, in particular melanoma: Leukaemia (HL-60), Lung (EKVX), CNS (SF-295, SNB-19), Melanoma (MALME-3M, M14, SK-MEL-2, SK MEL-28, UACC-257), Ovarian (OVCAR-5, NCI/ADR-RES) and Breast (T-47D). 

Ketone 13 exerts a far more cytotoxic effect across all cell lines with consistent cell death evident at 10 µM as in the one dose screen (Table 2, LC50 column; Figures S13 and S14). Aside the exception of the leukaemia cell panel, all cells are killed by 100 µM of 13 which suggests a potent and common mechanism of action. The compound is exceptionally potent against the growth of A498 renal cancer cells with a GI50 of 386 nM and merits future investigation. In the design of compound 13, the Michael acceptor moiety is presumably involved in alkylation of the essential machinery of the cell and development of this is the subject of current work. 

In comparing the global effect of both 11 and 13 (Figures S13 and S16) it is evident that they affect cell growth by different mechanisms as would be expected from their structures. In specific, compound 11 has the potential for use in ovarian cancers given its exceptional toxicity against the OVCAR-3 and OVCAR-4 phenotypes (LC50 6.2 and <10 μM respectively; OVCAR-4 value assigned due to inflection of graph). OVCAR-3 and OVCAR-4 are both mutant p53 status but have individual profiles with OVCAR-3 having known upregulation of BRCA2-interacting transcriptional repressor and cyclin E1. OVCAR-4 in turn has been identified as a good model cell line for High Grade Serious Ovarian Cancer (HGSOC) which make these findings significant from a clinical perspective [33].

### 2.3. COMPARE Analysis of Compounds **11** and **13**

COMPARE analysis was performed on the NCI COMPARE algorithm in order to identify correlations with known anticancer agents within the NCI database. Both compound 11 and 13 were analysed and with a specific focus on two subsets of the database: synthetic compounds and mechanistic set. Compound 13 gave a number of correlations as high as 0.74 with the most relevant mechanistic hit being to Chrysanthemic acid (NSC 11779) but this must be seen in the overall non-specific profile of activity of 13 and its limited potency. 

However, on analysis of 11 in the synthetic compounds set, the top ranked correlation (0.6) is to SCH1473759 (NSC 761691) which has recently been reported as a picomolar inhibitor of Aurora kinases [34]. Again, while an important finding and a good match of cellular activity profile, this result must be seen in the context of potency with SCH1473759 having >100 the potency of 11 in the NCI assay (Figure S17). Compound 11 also gave rise to a correlation of 0.58 to Aclarubicin (Aclacinomycin A, NSC 208734) being the top hit of the mechanistic compound set. Aclarubicin has known topoisomerase inhibitory and recently reported histone eviction from open chromatin activity so despite the lack of evident topoisomerase activity of 11, this result merits further evaluation in respect of its interaction with DNA and DNA interacting enzymes [35].

## 3. Materials and Methods

Solvents were distilled prior to use as follows: dichloromethane was distilled from phosphorous pentoxide; ethyl acetate was distilled from potassium carbonate; ethanol and methanol were distilled from magnesium in the presence of iodine; toluene was distilled from sodium and benzophenone; hexane was distilled prior to use; tetrahydrofuran was freshly distilled from sodium and benzophenone. Diethyl ether was obtained pure from Riedel-de Haën. Organic phases were dried using anhydrous magnesium sulphate. All commercial reagents were used without further purification unless otherwise stated. All samples were confirmed as >95% pure by use of high resolution LCMS analysis.

Infrared spectra were recorded as a thin film on sodium chloride plates for liquids or potassium bromide (KBr) disc for solids on a Perkin Elmer Spectrum 100 FT-IR spectrometer.

^1^H (300 MHz) and ^13^C (75 MHz) NMR spectra were recorded on a Bruker Avance 300 NMR spectrometer. ^1^H (600 MHz) and ^13^C (150.9 MHz) NMR spectra were recorded on a Bruker Avance III 600 MHz NMR spectrometer equipped with a dual CH cryoprobe. All spectra were recorded at room temperature (~20 °C) in deuterated dimethylsulfoxide (DMSO-*d*_6_) were assigned using the DMSO-*d*_6_ peak as the reference peak. Chemical shifts (δH and δC) are expressed in parts per million (ppm) relative to the reference peak. Coupling constants (J) are expressed in Hertz (Hz). Splitting patterns in ^1^H NMR spectra are designated as s (singlet), br s (broad singlet), d (doublet), br d (broad doublet), t (triplet), q (quartet), dd (doublet of doublets), dt (doublet of triplets), ddd (doublet of doublet of doublets), ddt (doublet of doublet of triplets) and m (multiplet). 

Low resolution mass spectra were recorded on a Waters Quattro Micro triple quadrupole spectrometer (QAA1202) in electrospray ionisation (ESI) mode using 50% acetonitrile:water containing 0.1% formic acid as eluent. High resolution mass spectra (HRMS) were recorded on a Waters LCT Premier Time of Flight spectrometer (KD-160) in electrospray ionisation (ESI) mode using 50% acetonitrile:ater containing 0.1% formic acid as eluent. 

Melting points were measured in a uni-melt Thomas Hoover capillary melting point apparatus and are uncorrected. Thin layer chromatography (TLC) was carried out on precoated silica gel plates (Merck 60 PF254) or aluminium oxide TLC paltes (Sigma). Visualisation was achieved by UV light detection (254 nm).

**5-Methyl-6*H*-pyrido[4,3-*b*]carbazole-11-carboxylic acid 9.** 5-Methyl-6*H*-pyrido[4,3-*b*]carbazole-11-carbaldehyde **8** (92 mg, 0.353 mmol) in acetonitrile (9 mL) was treated with dimethylsulfoxide (0.03 mL, 0.423 mmol) and conc. sulfuric acid (0.3 mL, 0.622 mmol). A solution of sodium chlorite (48 mg, 0.530 mmol) in water (3 mL) was added dropwise and the reaction mixture was stirred at room temperature overnight. The reaction was quenched with sodium sulfite (22 mg, 0.177 mmol) in water (1 mL). The acetonitrile was evaporated to leave an aqueous solution, which was carefully adjusted to pH 5 with saturated aqueous sodium bicarbonate to precipitate the product. The mixture was cooled to 0 °C, filtered and washed with water (3 mL), to give 5-methyl-6*H*-pyrido[4,3-*b*]carbazole-11-carboxylic acid **9**. The orange solid was dried at 0.02 mbar for 24 h (71 mg, 73.2%). m.p. >300 °C; vmax/cm^−1^ (KBr): 3161 (NH), 3010 (OH broad), 1689 (C=O), 1648 (C=C arom.), 1597 (C=C arom.), 1488, 1463, 1417, 1241 (C-O stretch), 1109; δH (300 MHz, DMSO-*d*_6_): 2.88 [3H, s, C(5)CH_3_], 7.26 [1H, t, *J* = 7.9, C(9)H], 7.55–7.62 [2H, m, C(7)H, C(8)H], 8.03 [1H, d, *J* = 5.9, C(3)H], 8.21 [1H, d, *J* = 7.8, C(10)H], 8.47 [1H, d, *J* = 5.9, C(4)H], 9.43 [1H, s, C(1)H], 11.64 [1H, s, N(6)H]; δC (75.5 MHz, DMSO-*d*_6_): 12.3 [3H, s, C(5)CH_3_], 111.5 [CH, C(7)H], 113.3 (C, aromatic C), 117.7 [CH, C(3)H], 118.5 (C, aromatic C), 120.0 [CH, C(9)H], 120.3 (C, aromatic C), 122.3 (C, aromatic C), 123.1 [CH, C(10)H], 124.9 (C, aromatic C), 129.1 [CH, C(8)H], 132.2 (C, aromatic C), 136.1 [CH, C(4)H], 141.9 (C, aromatic C), 143.1 (C, aromatic C), 147.9 [CH, C(1)H], 168.8 [C, C(11)COOH]; m/z (ESI+): 277 [(M+H)^+^ 40%], 115 (100%); HRMS (ESI+): Exact mass calculated for [C_17_H_13_N_2_O_2_]^+^ 277.0977. Found 277.0977.

**5-Methyl-6*H*-pyrido[4,3-*b*]carbazole-11-carbonylchloride 10.** 5-Methyl-6*H*-pyrido[4,3-*b*]carbazole-11-carboxylic acid **9** (99 mg, 0.358 mmol) in dichloromethane (50 mL), under N_2_, was treated with oxalyl chloride (0.04 mL, 0.459 mmol). Slight fizzing was observed on addition and mixture was stirred at room temperature for 20 h. The bright orange suspension was evaporated under reduced pressure. IR analysis showed the carbonyl peak at 1785 cm^−1^ indicating acid chloride formation.

***N*-Benzyl-5-methyl-6*H*-pyrido[4,3-*b*]carbazole-11-carboxamide 11.** 5-Methyl-6*H*-pyrido[4,3-*b*]carbazole-11-carboxylic acid **9** (99 mg, 0.358 mmol) in dichloromethane (50 mL), under N_2_, was treated with oxalyl chloride (0.04 mL, 0.459 mmol) and stirred at room temperature for 20 h. The mixture was evaporated under reduced pressure, cooled to 0 °C under N_2_, and treated drop-wise with benzylamine (2 mL, 18.3 mmol). After stirring for 1 h, diethyl ether (40 mL) was added, the mixture was cooled and filtered to give a cream solid (220 mg) containing the desired product and residual benzylamine. This was stirred with diethyl ether and decanted (3 × 40 mL). Purification by column chromatography, eluting with dichloromethane:methanol (100:0–95:5), gave product **11** as a yellow solid (39 mg, 30%). m.p. 294–296 °C; vmax/cm^−1^ (KBr): 3159 (NH), 3051 (CH) 1731 (C=O), 1621 (C=C arom.), 1600 (C=C arom.), 1542, 1466, 1410, 1245; δH (600 MHz, DMSO-*d*_6_): 2.79 [3H, s, C(5)CH_3_], 4.65–4.70 [2H, m, CONHCH_2_], 6.98 [1H, t, *J* = 7.4, C(9)H], 7.28 [1H, t, *J* = 7.2, N-benzyl-C(4)H], 7.36 [2H, t, *J* = 7.3, *N*-benzyl-C(2)H, C(6)H], 7.43–7.50 [4H, m, C(7)H, C(8)H, *N*-benzyl-C(3)H, C(5)H], 7.72 [1H, d, *J* = 7.8, C(10)H], 7.93 [1H, d, *J* = 5.9, C(3)H], 8.37 [1H, d, *J* = 5.1, C(4)H], 9.19 [1H, s, C(1)H], 9.41 [1H, t, *J* = 5.9, C(11)CONH], 11.46 [1H, s, N(6)H]; δC (150.9 MHz, DMSO-*d*_6_): 12.2 [CH_3_, C(5)CH_3_], 43.1 [CH_2_, C(11)CONHCH_2_], 110.9 (CH, aromatic CH), 111.3 (C, aromatic C), 116.0 [CH, C(3)H], 119.1 [CH, C(9)H], 119.8 (C, aromatic C), 120.9 (C, aromatic C), 121.1 (C, aromatic C), 122.8 [CH, C(10)H], 127.1 [CH, N-benzyl-C(4)H], 127.2 (C, aromatic C), 127.9 (CH, aromatic CH), 128.0 (CH, aromatic CH), 128.2 (CH, aromatic CH), 128.4 (CH, aromatic CH), 128.5 (CH, aromatic CH), 131.7 (C, aromatic C), 138.9 (C, aromatic C), 140.4 (C, aromatic C), 140.7 [CH, C(4)H], 142.7 (C, aromatic C), 150.1 [CH, C(1)H], 167.1 [C, C(11)CONH]; m/z (ESI+): 366 [(M+H^+^), 100%]; HRMS (ESI+): Exact mass calculated for [C_24_H_20_N_3_O]^+^ 366.1606. Found 366.1615.

**Methyl 5-methyl-6*H*-pyrido[4,3-*b*]carbazole-11-carboxylate 12.** 5-Methyl-6*H*-pyrido[4,3-*b*]carbazole-11-carboxylic acid **9** (99 mg, 0.358 mmol) in dimethylformamide (8 mL) under N_2_, was heated gently until the acid dissolved. Carbonyldiimidazole (70 mg, 0.430 mmol) was added and the solution was heated at 120 °C for 4 h. The mixture was cooled to 0 °C and diethyl ether (20 mL) was added. The resulting precipitate was filtered and washed (diethyl ether 10 mL). The solid was cooled to 0 °C and treated dropwise with methanol (10 mL). The mixture was stirred at r.t. for 1 h and heated to reflux for 5 h. On cooling, a precipitate formed which was filtered to give the methyl ester, methyl 5-methyl-6*H*-pyrido[4,3-*b*]carbazole-11-carboxylate **12** (14 mg, 14%). m.p. 247–250 °C; vmax/cm^−1^ (KBr): 3147 (NH), 3008 (CH), 1742 (C=O), 1634 (C=C arom.), 1589 (C=C arom.), 1457, 1422, 1246 (C-O stretch), 1095; δH (300 MHz, DMSO-*d*_6_): 2.89 [3H, s, C(5)CH_3_], 4.22 [3H, s, C(11)COOCH_3_], 7.27 [1H, overlapping ddd, *J* = 8.0, 5.8, 2.3, C(9)H], 7.57–7.63 [2H, m, C(7)H, C(8)H], 7.99 [1H, d, *J* = 8.0, C(10)H], 8.04 [1H, d, *J* = 6.1, C(3)H], 8.50 [1H, br s, C(4)H], 9.38 [1H, br s, C(1)H], 11.73 [1H, s, N(6)H]; δC (150.9 MHz, DMSO-*d*_6_): 12.4 [CH_3_, C(5)CH_3_], 53.1 [CH_3_, C(11)COOCH_3_], 111.3 [CH, C(7)H or C(8)H], 113.7 (C, aromatic C), 116.2 [CH, C(3)H], 119.6 [CH, C(9)H], 120.2 (C, aromatic C), 121.3 (C, aromatic C), 121.8 (C, aromatic C), 122.5 [CH, C(10)H], 128.7 [CH, C(7)H or C(8)H], 131.6 (C, aromatic C), 140.2 (C, aromatic C), 140.9 [CH, C(4)H], 143.1 (C, aromatic C), 149.8 [CH, C(1)H], 168.4 [C, C(11)C=O]; m/z (ESI+): 291 [(M+H)^+^, 100%]. HRMS (ESI+): Exact mass calculated for [C_18_H_15_N_2_O_2_]^+^ 291.1134. Found 291.1121.

**(*E*)-4-(5-methyl-6*H*-pyrido[4,3-*b*]carbazol-11-yl)but-3-en-2-one 13.** A stirred solution of 5-methyl-6*H*-pyrido[4,3-*b*]carbazole-11-carbaldehyde **8** (300 mg, 1.15 mmol) in acetone (70 mL) was treated drop-wise with potassium permanganate (364 mg, 2.30 mmol) in water (70 mL). The mixture was stirred at r.t. for 1 h and heated to reflux for 22 h. Sodium bicarbonate (244 mg, 2.30 mmol) was added and stirred for 20 min. The mixture was filtered through celite and washed with water (100 mL) and acetone (200 mL). The filtrate was concentrated under reduced pressure to remove the acetone and then acidified to pH 5 with 20% aqueous HCl. The aqueous layer was extracted with chloroform:methanol (90:10, 3 × 100 mL). Organic extracts were combined and washed with water (1 × 100 mL) and brine (1 × 100 mL), dried over magnesium sulphate and evaporated under reduced pressure to give an orange solid (300 mg). Analysis showed that the desired carboxylic acid **9** had not formed but instead the condensation product (*E*)-4-(5-methyl-6*H*-pyrido[4,3-*b*]carbazol-11-yl)but-3-en-2-one **13** (87.0%). m.p. 263–265 °C; vmax/cm^−1^ (KBr): 3149(NH), 3087 (CH), 2982 (asymm. CH_3_ stretch), 2883 (symm. CH_3_ stretch), 1664 (C=O), 1620 (C=C), 1592 (C=C arom.), 1464, 1404, 1383, 1243; δH (300 MHz, DMSO-*d*_6_): 2.61 [3H, s, COCH_3_], 2.84 [3H, s, C(5)CH_3_], 6.75 [1H, d, *J* = 16.6, C(11)CH=CH], 7.25 [1H, overlapping ddd, *J* = 8.0, 6.6, 1.5, C(9)H], 7.53–7.61 [2H, m, C(7)H, C(8)H], 7.98 [1H, dd, *J* = 6.1, 0.7, C(3)H], 8.14 [1H, d, *J* = 8.0, C(10)H], 8.47 [1H, d, *J* = 6.1, C(4)H], 8.59 [1H, d, *J* = 16.6, C(11)CH=CH], 9.58 [1H, s, C(1)H], 11.57 [1H, s, N(6)H]; δC (75.5 MHz, DMSO-*d*_6_): 12.2 [CH_3_, C(5)CH_3_], 27.8 (CH_3_, COCH_3_), 111.1 [CH, C(7)H], 111.7 (C, aromatic C), 115.9 [CH, C(3)H], 119.4 [CH, C(9)H], 120.5 (C, aromatic C), 121.9 (C, aromatic C), 122.4 (C, aromatic C), 123.6 [CH, C(10)H], 126.0 (C, aromatic C), 127.9 [CH, C(8)H], 132.3 (C, aromatic C), 135.9 [CH, C(11)CH=CH], 138.4 [CH, C(11)CH=CH], 140.2 (C, aromatic C), 140.9 [CH, C(4)H], 143.0 (C, aromatic C), 150.1 [CH, C(1)H], 197.8 (C, C=O); m/z (ESI+): 301[(M+H)+ 100%]; HRMS (ESI+): Exact mass calculated for [C_20_H_17_N_2_O]^+^ 301.1341. Found 301.1348.

**(*E*)-*N*-(4-(5-methyl-6*H*-pyrido[4,3-*b*]carbazol-11-yl)but-3-en-2-yl)formamide 14.** (*E*)-4-(5-Methyl-6*H*-pyrido[4,3-*b*]carbazol-11-yl)but-3-en-2-one **13** (91 mg, 0.303 mmol), formamide (0.2 mL, 5.03 mmol) and formic acid (0.1 mL, 2.65 mmol) were heated to 150 °C for 1.5 h, at which point TLC analysis indicated consumption of the starting material. On cooling, saturated aqueous sodium bicarbonate (8 mL) was added and extraction was attempted with dichloromethane:methanol (90:10), however the organic extracts contained no material. The aqueous layer was treated with 20% aqueous HCl to pH 1, stirred for 20 min and then adjusted to pH 10 with 20% aqueous NaOH. The aqueous layer was extracted with dichloromethane–methanol (90:10 3 × 30 mL and 80:20 4 × 30 mL), dried and evaporated under reduced pressure to give an orange solid (64 mg). Purification by column chromatography on alumina, eluting with dichloromethane:methanol (97:3–90:10) gave three fractions. The largest of these (34 mg) was repurified by chromatography on silica to give an orange solid (19 mg). This was found to be the formamide, (*E*)-*N*-(4-(5-methyl-6*H*-pyrido[4,3-*b*]carbazol-11-yl)but-3-en-2-yl)formamide **14**. δH (300 MHz, DMSO-*d*_6_): 1.47 [3H, d, *J* = 6.9, C(11)CH=CH–CH(CH_3_)], 2.83 [3H, s, C(5)CH_3_], 4.88–4.95 [1H, m, C(11)CH=CH–CH(CH_3_)], 6.14 [1H, dd, *J* = 16.2, 5.6, C(11)CH=CH], 7.21 [1H, overlapping ddd, *J* = 8.0, 6.7, 1.4, C(9)H], 7.36 [1H, d, *J* = 16.5, C(11)CH=CH], 7.52 [1H, td, *J* = 7.4, 1.1, C(8)H], 7.56–7.59 [1H, m, C(7)H], 7.99 [1H, br s, NHCHO], 8.23 [1H, br s, C(3)H], 8.33 [1H, d, *J* = 7.7, C(10)H], 8.43 [1H, br s, NHCHO], 8.54 [1H, d, *J* = 7.5, C(4)H], 9.58 [1H, br s, C(1)H], 11.51 [1H, s, N(6)H]; m/z (ESI+): 330 [(M+H)^+^ 60%], 169 (100%); HRMS (ESI): Exact mass calculated for [C_21_H_20_N_3_O]^+^ 330.1606. Found 330.1619.

**9-Formyl-5-methyl-6*H*-pyrido[4,3-*b*]carbazole-11-carboxylic acid 15.** 5-Methyl-6*H*-pyrido[4,3-*b*]carbazole-11-carboxylic acid **9** (497 mg, 1.80 mmol) in trifluoroacetic acid (60 mL), was treated with hexamethylenetetramine (2.522 g, 18.0 mmol) portionwise over 5 min and heated to reflux for 25 min. On cooling, the reaction mixture was concentrated to approx. one quarter volume, water (30 mL) was added and the solution was transferred to a large conical flask (500 mL). The solution was cooled to 0 °C and neutralized with solid sodium bicarbonate while stirring vigorously. The mixture was stirred at 0 °C for 1 h, readjusted to pH 7 and filtered to give a brown solid which was dried at 0.02 mbar for 2 days (449 mg, 81.9%). m.p. 315–317 °C; vmax/cm^−1^ (KBr): 3069 (OH broad), 1677 (C=O × 2, broad), 1583 (C=C arom.), 1472, 1404, 1349, 1242 (C-O stretch), 1128, 808; δH (600 MHz, DMSO-*d*_6_): 2.88 [3H, s, C(5)CH_3_], 7.72 [1H, d, *J* = 8.3, C(7)H], 8.04 [1H, d, *J* = 6.0, C(3)H], 8.10 [1H, d, *J* = 8.4, C(8)H], 8.50 [1H, d, *J* = 6.0, C(4)H], 8.80 [1H, s, C(10)H], 9.51 [1H, s, C(1)H], 10.04 [1H, s, C(9)CHO], 12.14 [1H, s, N(6)H]; δC (150.9 MHz, DMSO-*d*_6_): 12.3 [3H, s, C(5)CH_3_], 111.4 [CH, C(7)H], 113.0 (C, aromatic C), 116.2 [CH, C(3)H], 119.7 (C, aromatic C), 120.4 (C, aromatic C), 121.3 (C, aromatic C), 125.7 [CH, C(10)H], 128.5 (C, aromatic C), 129.8 [CH, C(8)H], 129.9 (C, aromatic C), 132.4 (C, aromatic C), 140.8 [CH, C(4)H], 141.1 (C, aromatic C), 146.9 (C, aromatic C), 150.7 [CH, C(1)H], 169.2 [C, C(11)COOH], 191.6 [CH, C(9)CHO]; m/z (ESI+): 305 [(M+H)^+^ 70%], 155 (40%), 64 (100%); HRMS (ESI+): Exact mass calculated for [C_18_H_13_N_2_O_3_]^+^ 305.0926. Found 305.0940.

**Benzylammonium 9-((benzylimino)methyl)-5-methyl-6H-pyrido[4,3-b]carbazole-11-carboxylate 16.** A suspension of 9-formyl-5-methyl-6*H*-pyrido[4,3-*b*]carbazole-11-carboxylic acid **15** (194 mg, 0.638 mmol) in dichloromethane (65 mL), under N_2_, was treated with oxalyl chloride (0.08 mL, 0.917 mmol) and stirred at room temperature overnight. Additional oxalyl chloride (0.08 mL, 0.917 mmol), was added and the reaction was stirred for 4 h. The mixture was evaporated under reduced pressure, cooled to 0 °C under N_2_, and treated drop-wise with benzylamine (2 mL, 18.3 mmol). After stirring for 1 h, diethyl ether (40 mL) was added, the mixture was cooled and filtered to give a yellow solid. Crude analysis showed this to contain residual benzylamine. Recrystallisation from dichloromethane followed by a second recrystallisation from methanol gave product still containing benzylamine in 1:1 ratio with product. To investigate whether an amide or imine had formed, the product (27 mg) was dissolved in dichloromethane:methanol (90:10, 10 mL) and washed with 1M HCl (10 mL). The organic layer was dried and evaporated under reduced pressure. NMR and MS analysis showed that the compound had hydrolysed to the starting material **15**, indicating imine rather than amide formation. Full analysis (along with subsequent reduction of the imine) confirmed the product as the imine salt, benzylammonium 9-((benzylimino)methyl)-5-methyl-6*H*-pyrido[4,3-*b*]carbazole-11-carboxylate **16** (208 mg, 82.9%). m.p. 241–243 °C; vmax/cm^−1^ (KBr): 3029 (NH), 2864 (OH, broad), 1598 (C=O), 1572 (C=C arom.), 1494, 1450, 1402, 1347, 1278, 1244 (C-O stretch); δH (300 MHz, DMSO-*d*_6_): 2.81 [3H, s, C(5)CH_3_], 4.06 [2H, s, benzylammmonium-CH_2_], 4.74 [2H, s, C(9)CH=NCH_2_], 7.23–7.29 [1H, m, iminobenzyl-C(4)H], 7.33–7.42 [7H, m, iminobenzyl-C(2)H, C(3)H, C(5)H, C(6)H, benzylammmonium-C(3)H, C(4)H, C(5)H], 7.48 [2H, dd, *J* = 7.7, 1.7, benzylammmonium-C(2)H, C(6)H], 7.56 [1H, d, *J* = 8.3, C(7)H], 7.91 [1H, dd, *J* = 6.1, 0.7, C(3)H], 7.97 [1H, dd, *J* = 8.4, 1.6, C(8)H], 8.38 [1H, d, *J* = 6.1, C(4)H], 8.52 [1H, s, C(9)CH=N], 8.73 [1H, d, *J* = 1.1, C(10)H], 9.50 [1H, s, C(1)H], 11.64 [1H, s, N(6)H]; δC (75.5 MHz, DMSO-*d*_6_): 12.0 [CH_3_, C(5)CH_3_], 42.3 [CH_2_, benzylammonium CH_2_], 64.1 [CH_2_, C(9)CH=N–CH_2_], 110.6 (CH, aromatic CH), 115.7 (CH, aromatic CH), 119.2 (C, aromatic C), 119.4 (C, aromatic C), 122.2 (C, aromatic C), 124.3 (CH, aromatic CH), 125.1 (C, aromatic C), 126.7 (CH, aromatic CH), 127.3 (C, aromatic C), 127.9 (CH, 2 × aromatic CH), 128.2 (CH, aromatic CH), 128.3 (CH, 2 × aromatic CH), 128.5 (CH, 2 × aromatic CH), 128.6 (CH, aromatic CH), 128.7 (CH, 2 × aromatic CH), 131.3 (C, aromatic C), 132.1 (C, aromatic C), 134.6 (C, aromatic C), 139.9 (CH, aromatic CH), 140.5 (CH, aromatic CH), 141.1 (C, aromatic C), 144.3 (C, aromatic C), 144.5 (C, aromatic C), 152.0 (CH, aromatic CH), 162.2 (C, C=O); m/z (ESI+): 394 [(M+H)^+^ 20%], 305 (20%), 108 (100%); HRMS (ESI+): Exact mass calculated for [C_25_H_20_N_3_O_2_]^+^ 394.1556. Found 394.1560.

**9-((Benzylamino)methyl)-5-methyl-6*H*-pyrido[4,3-*b*]carbazole-11-carboxylic acid 17.** A solution of benzylammonium 9-((benzylimino) methyl)-5-methyl-6*H*-pyrido[4,3-*b*]carbazole-11-carboxylate **16** (88 mg, 0.224 mmol) in absolute ethanol (12 mL) was treated with sodium cyanoborohydride (21 mg, 0.338 mmol) and heated to reflux for 4.5 h. The solution was evaporated under reduced pressure and water (10 mL) was added. The mixture was stirred for 15 min, cooled and filtered. The orange solid was washed with water (5 mL) and diethyl ether (10 mL) and dried at 0.1 mbar for 24 h (56 mg, 62.9%). m.p. >300 °C (without melting); vmax/cm^−1^ (KBr): 3370 (NH), 3188 (OH, broad), 3056 (CH), 1596 (C=O), 1565 (C=C arom.), 1482, 1403, 1348, 1247 (C-O stretch); δH (600 MHz, DMSO-*d*_6_): 2.73 [3H, s, C(5)CH_3_], 3.89 [2H, s, one of CH_2_], 4.04 [2H, s, one of CH_2_], 7.21–7.26 [3H, m, *N*-benzyl-C(3)H, C(4)H, C(5)H], 7.39–7.40 [2H, m, N-benzyl-C(2)H, C(6)H], 7.46 [1H, d, *J* = 7.3, C(7)H], 7.53 [1H, d, *J* = 7.5, C(8)H], 7.82 [1H, d, *J* = 5.6, C(3)H], 8.29 [1H, d, *J* = 5.6, C(4)H], 8.42 [1H, s, C(10)H], 9.44 [1H, s, C(1)H], 11.39 [1H, s, N(6)H]; δC (150.9 MHz, DMSO-*d*_6_): 12.0 [CH_3_, C(5)CH_3_], 50.3 (CH_2_), 51.4 (CH_2_), 108.3 (C, aromatic C), 110.4 [CH, C(7)H], 115.6 [CH, C(3)H], 119.1 (C, aromatic C), 119.3 (C, aromatic C), 122.4 (C, aromatic C), 124.5 [CH, C(10)H], 127.7 [CH, *N*-benzyl-C(4)H], 128.1 (CH, C(8)H], 128.3 [CH, *N*-benzyl-C(3)H, *N*-benzyl-C(5)H], 129.0 [CH, *N*-benzyl-C(2)H, *N*-benzyl-C(6)H], 129.1 (C, aromatic C), 132.0 (C, aromatic C), 133.7 (C, aromatic C), 136.0 (C, aromatic C), 140.2 [CH, C(4)H], 141.3 (C, aromatic C), 142.2 (C, aromatic C), 152.2 [CH, C(1)H], 170.8 (C, C=O); m/z (ESI+): 396 [(M+H)^+^ 80%], 306 (100%); HRMS (ESI+): Exact mass calculated for [C_25_H_22_N_3_O_2_]^+^ 396.1712. Found 396.1729.

**Topoisomerase II decatenation assay.** The decatenation assay kit was obtained from Inspiralis, Norwich Bioincubator, Norwich Research Park, Colney, Norwich, UK. The kit comprised of the following: assay buffer (supplied as 10× stock) containing 50 mM Tris.HCl (pH 7.5), 125 mM NaCl, 10 mM MgCl2, 5 mM DTT and 100 µg/mL albumin; dilution buffer containing 50 mM Tris. HCl (pH 7.5), 100 mM NaCl, 1 mM DTT, 0.5 mM EDTA, 50% (v/v) glycerol, 50 µg/mL albumin; ATP 30 mM; kDNA (100 ng/µl); 10 U/µL human topoisomerase II in dilution buffer; 5× stop buffer containing 2.5% SDS, 15% Ficoll-400, 0.05% bromophenol blue, 0.05% xylene cyanol and 25 mM EDTA. Tris-acetate-EDTA buffer (supplied as 10X buffer) and agarose were obtained from Sigma Life Sciences (Dublin, Ireland) and Safe View Stain was supplied by NBS Biologicals, Cambridgeshire, England.

The topo II decatenation assay protocol involved initial incubation of each inhibitor candidate (100 µM) along with a stock solution containing water, ATP, assay buffer, kDNA obtained from the mitochondrial DNA of Crithidia fasciculate, and topo II, at 37 °C for 1 h. Following addition of stop buffer, agarose DNA gel electrophoresis was run at 50 V for 2 h using a Consort EV243 power pack, to determine the relative amounts of decatenated DNA bands obtained in each compound lane. Positive (water), as well as negative controls (ellipticine) were incorporated in order to validate the results of each run. The resulting gels were viewed under UV light using a DNR Bio-Imaging System and photographed using GelCapture software.

**NCI-60 Anti-cancer screening.** Tested compounds were initially solubilised in DMSO, diluted into RPMI 1640 and 5% fetal bovine serum/L-glutamine, and added to 96-well plates containing cell lines previously cultured for 24 h. After 48-h incubation, the media were removed, and the cells were fixed and stained with sulforhodamine B to determine overall percent growth/total protein content. Unbound dye was removed with five washes of 1% acetic acid, and the plates were allowed to air dry. The dye was then resolubilised in Tris buffer, and the colorimetric absorbance was measured (515 nm). Growth inhibition was measured relative to the response generated from proliferating cells cultured under identical conditions for 48 h. In the five dose study, serial 5 × 10-fold dilution from an initial DMSO stock solution was performed, prior to incubation at each individual concentration (10 nM, 100 nM, 1 µM, 10 µM and 100 µM).

Using seven absorbance measurements (time zero (Tz), control growth (C), and test growth in the presence of drug at the five concentration levels (Ti)), the percentage growth was calculated at each of the drug concentrations levels. Percentage growth inhibition was calculated as:

[(Ti−Tz)/(C−Tz)] × 100 for concentrations for which Ti ≥ Tz
[(Ti−Tz)/Tz] × 100 for concentrations for which Ti < Tz.

Three dose response parameters were calculated for each experimental agent. Growth inhibition of 50% (GI50) was calculated from [(Ti−Tz)/(C−Tz)] × 100 = 50, which is the drug concentration resulting in a 50% reduction in the net protein increase (as measured by Sulforhodamine B staining) in control cells during the drug incubation. The drug concentration resulting in total growth inhibition (TGI) was calculated from Ti = Tz. The LC50 (concentration of drug resulting in a 50% reduction in the measured protein at the end of the drug treatment as compared to that at the beginning) indicating a net loss of cells following treatment was calculated from [(Ti−Tz)/Tz] × 100 = −50. Values were calculated for each of these three parameters if the level of activity was reached; however, if the effect was not reached or was exceeded, the value for that parameter was expressed as greater or less than the maximum or minimum concentration tested [36,37]. Data from one dose experiments pertains to the percentage growth at 10 µM.

**COMPARE analysis.** COMPARE analysis was conducted using the private access system provided by the National Cancer Institute (https://dtp.cancer.gov/databases_tools/compare.htm). Seed compounds were analysed using a number of target sets: synthetic compounds, mechanistic set, standard agents, marketed drugs and diversity set. While the minimum correlation was set to 0.4, correlations of less than 0.5 were discounted. All other criteria were unchanged. Experiments that were carried out at different concentrations to the seed compound were ignored unless the concentration deviated by ±0.1, as was usual for older testing methods. COMPARE analysis was conducted solely on five dose data for compounds **11** and **13** [38].

## 4. Conclusions

Novel ellipticines substituted at the 11-position are described and their activity evaluated against topoisomerase II and cell growth in the National Cancer Institute’s 60 cell line screening platform. Two of the novel compounds (**13** and **16**) show limited promise as topoisomerase II inhibitors at high concentrations (>10 µM). On evaluation of anticancer effect at the NCI, compounds **11** and **13** show real promise as future leads. Benzylamide **11** and unsaturated ketone **13** appear to have different modes of action in their cellular effects with broad cytotoxicity seen for compound **13** but some selectivity of cellular response seen for compound **11**. COMPARE analysis identified a potential target for compound **11** in Aurora kinase due to correlation of 0.6 with the known inhibitor SCH1473759. This will be investigated further in the future by synthesis of a series of ellipticine 11-amides to probe if this activity can be tuned further.

## Figures and Tables

**Table 1:** 11-Substituted ellipticine topoisomerase activity and National Cancer Institute (NCI) mean growth percent (single dose).

	R^1^ 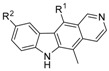 R^2^		Topo II Inhibition ^a^	NSC No	NCI Mean Growth %
**8**	CHO	H	−		Not tested
**9**	COOH	H	−	762124	99.92
**11**	CONHCH_2_Ph	H	−	762144	21.22
**13**	CH=CH-C(O)Me	H	+	762123	17.83
**15**	COOH	CHO	−	762141	95.56
**16**	COO^− +^NH_3_CH_2_Ph	CH=NCH_2_Ph	+	762142	106.19
**17**	COOH	CH_2_NHCH_2_Ph	−	762143	101.72

^a^ R^1^ = C-9 substituent; R^2^ = C11 substituent (+) Inhibition observed at 100 µM; (−) no activity observed at 100 µM.

**Table 2:** Selected GI50 and LC50 of the NCI 60 cell line panel for compounds **11** and **13** ^1^.

Cell Line	Cancer Subtype	11		13	
		GI50	LC50	GI50	LC50
HOP-62	Lung	2.15	>100	1.77	26.0
SW-620	Colon	2.86	>100	1.65	44.0
SNB-75	CNS	2.05	>100	2.65	34.8
OVCAR-3	Ovarian	2.33	<10	2.53	28.0
OVCAR-4	Ovarian	1.71	6.19	2.88	41.5
786-0	Renal	2.79	72.0	2.79	29.8
A498	Renal	50.5	>100	0.386	7.48
UO-31	Renal	2.73	>100	1.25	33.8
MCF7	Breast	2.71	>100	1.74	52.3
MDA-MB-231/ATCC	Breast	2.43	>100	1.74	41.3
HS578T	Breast	2.61	>100	1.96	48.4

^1^ data reported in µM values from NCI 60 cell line five dose assay; GI50: Growth Inhibition 50%; LC50: Lethal concentration 50%.

## Supplementary Materials

The following are available online at https://www.mdpi.com/1424-8247/12/2/90/s1: Figures S1–S3, ^1^H NMRs of relevant compounds; Figure S4, Three-fold dilution topoisomerase II inhibition assay of compounds **13** and **16**; Figures S5–S10, One dose NCI 60 cancer cell growth data (10 µM); Figures S11–S13, Five dose NCI 60 cancer cell growth data with GI50, TGI and LC50 data for compound **11**; Figures S14–S16, Five dose NCI 60 cancer cell growth data with GI50, TGI and LC50 data for compound **13**; Figure S17 COMPARE Analysis data for compound 13 in direct comparison with SCH1473759 (NSC761691), an Aurora kinase inhibitor.

## Abbreviations

The following abbreviations are used in this manuscript:

AKT	Protein Kinase B
CK2	Casein Kinase 2
GI50	Growth Inhibition 50%
LC50	Lethal concentration 50%
NCI	National Cancer Institute
NSC	numeric identifier for substances submitted to the National Cancer Institute (NCI)
Topo	Topoisomerase
SAR	Structure Activity Relationship
