# Supplementary material for: Novel 11-Substituted Ellipticines as Potent Anticancer Agents with Divergent Activity against Cancer Cells
Source: Pharmaceuticals (Basel). 2019 Jun 14;12(2):90. doi: 10.3390/ph12020090 (PMC6631919; doi:10.3390/ph12020090)

Novel 11-substituted ellipticines as potent anticancer agents with divergent activity against cancer cells

Charlotte M. Miller[1], Elaine C. O'Sullivan[1] and Florence O. McCarthy[1*]

[1] School of Chemistry, Analytical and Biological Chemistry Research Facility, University College Cork, Western Road, Cork, Ireland

* Correspondence: f.mccarthy@ucc.ie; Tel.: +353-21-4901695

Figures S1-S3	^{1}H NMRs of relevant compounds
Figure S4	Three-fold dilution topoisomerase II inhibition assay of compounds **13** and **16**
Figure S5-S10	One dose NCI 60 cancer cell growth data (10μM)
Figure S11-S13	Five dose NCI 60 cancer cell growth data with GI50, TGI and LC50 data for compound **11**
Figure S14-S16	Five dose NCI 60 cancer cell growth data with GI50, TGI and LC50 data for compound **13**
Figure S17:	COMPARE Analysis data for compound **13** in direct comparison with SCH1473759 (NSC761691), an Aurora kinase inhibitor.

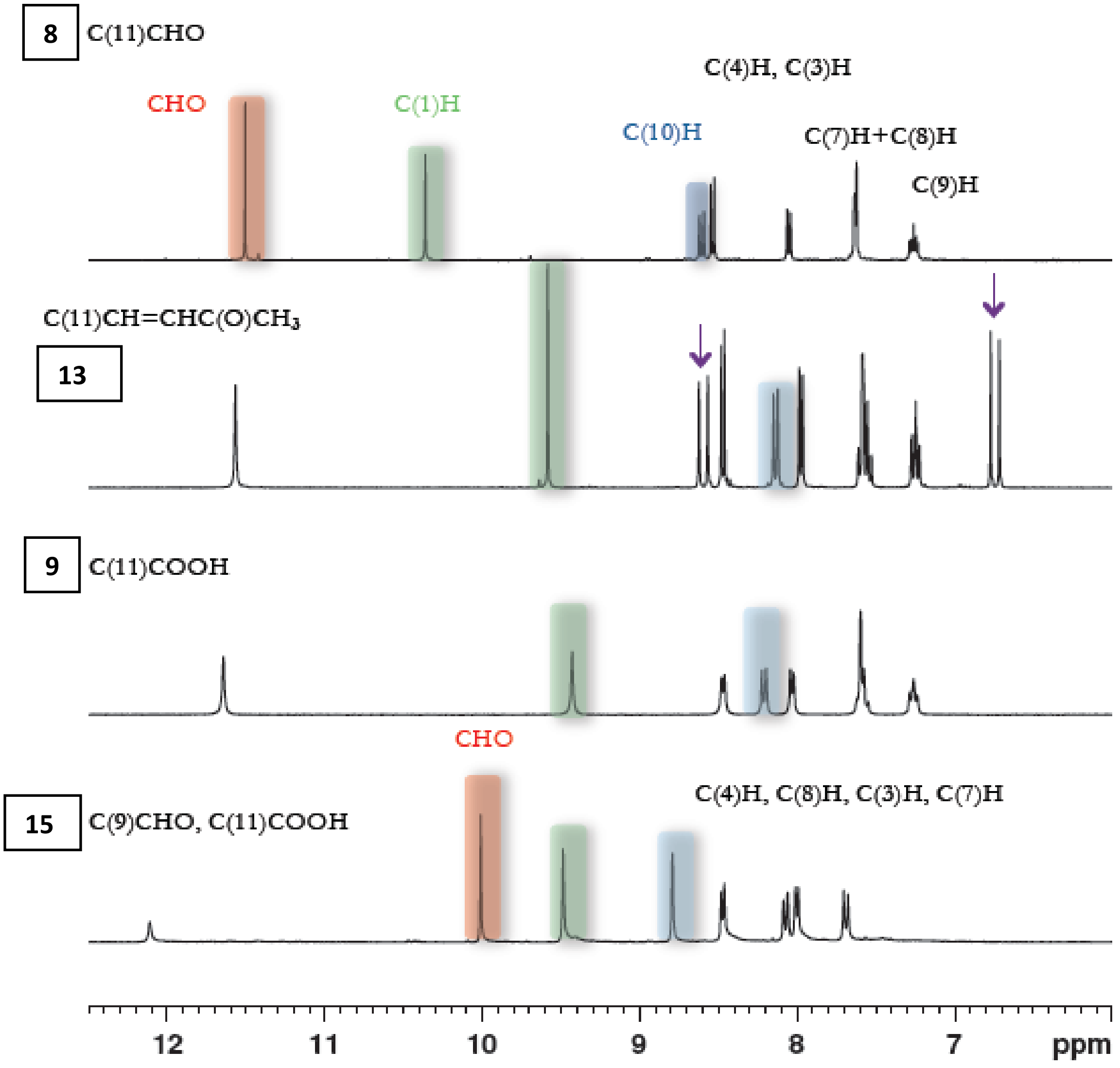

Figure S1: Stacked ^{1}H NMR plots of the aromatic regions of 11-substituted Ellipticines 8, 9, 13, and 15 measured in DMSO-d_6 at 300MHz

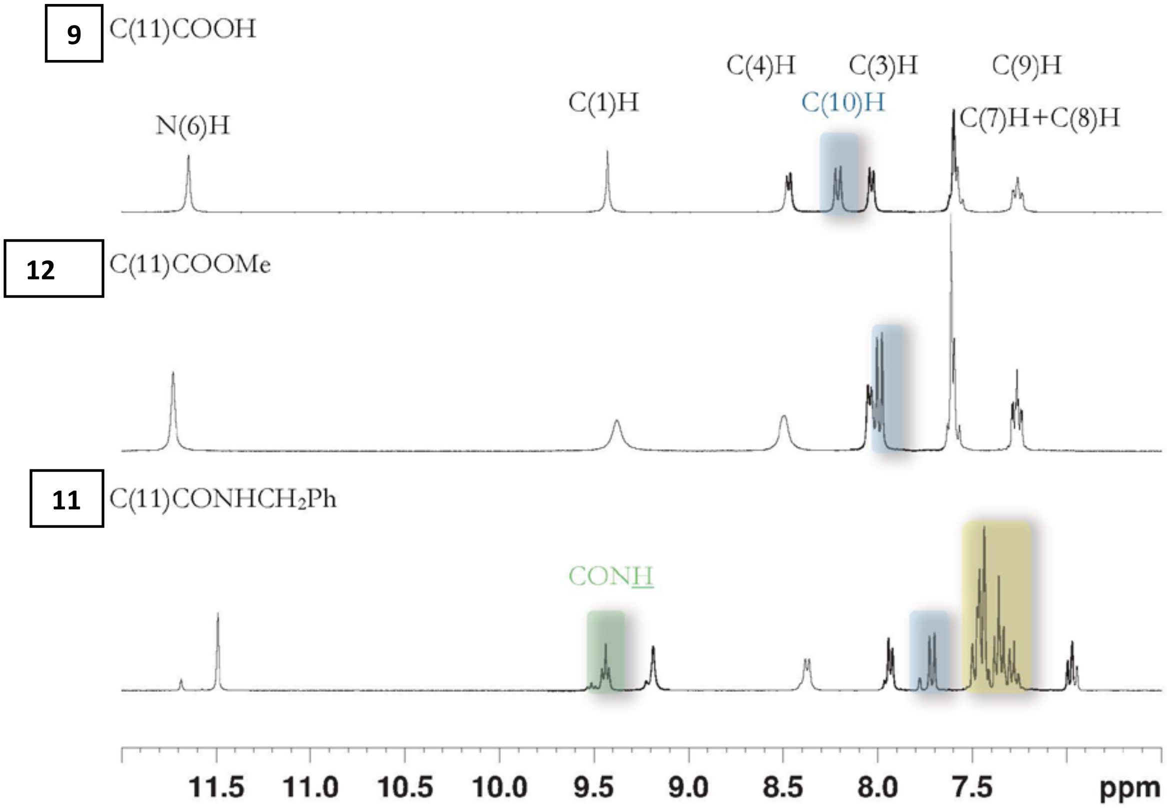

Figure S2: Stacked ^{1}H NMR plots of the aromatic regions of 11-substituted Ellipticines 9, 11 and 12 measured in DMSO-d_6 at 300MHz

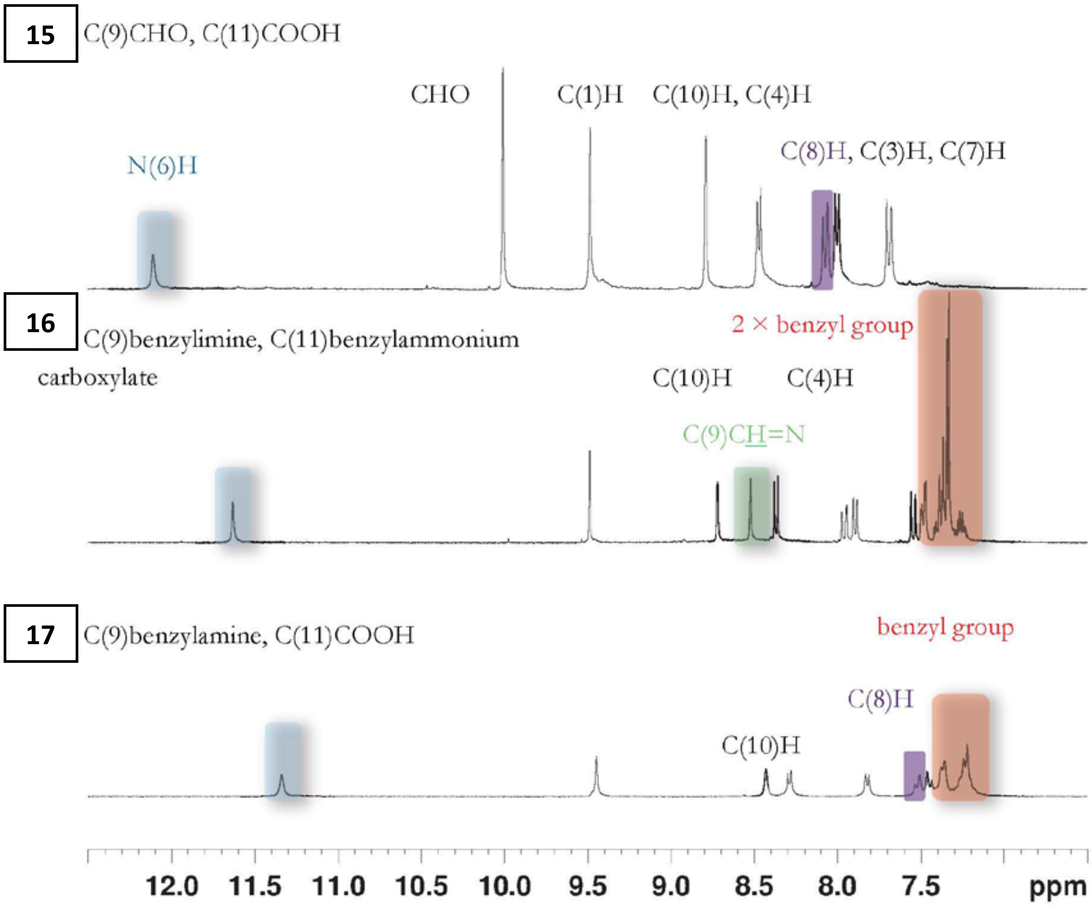

Figure S3: Stacked ^{1}H NMR plots of the aromatic regions of 11-substituted Ellipticines 15, 16 and 17 measured in DMSO-d_6 at 300MHz

Figure S4: Three fold dilution assay for compound 13 (lanes 1-3; 100-1uM) and 16 (lanes 4-6; 100-1uM); A &B are positive and negative control respectively.

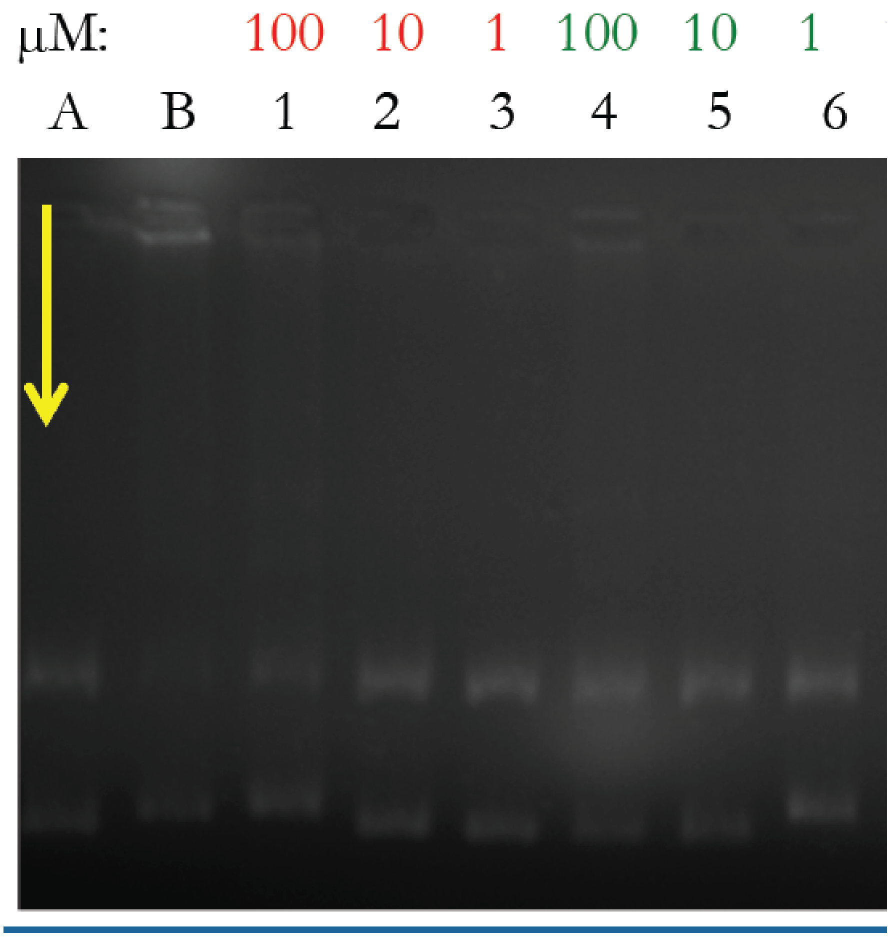

Figure S5: **One dose assay (10µM) NCI 60 cell lines growth data for compound 9**

Developmental Therapeutics Program	NSC: D-762124 / 1	Conc: 1.00E-5 Molar	Test Date: Nov 07, 2011
One Dose Mean Graph	Experiment ID: 1111OS59		Report Date: Dec 01, 2011

Panel/Cell Line	Growth Percent
Leukemia	
CCRF-CEM	105.71
HL-60(TB)	96.38
K-562	111.69
MOLT-4	96.71
RPMI-8226	101.79
SR	84.86
Non-Small Cell Lung Cancer	
A549/ATCC	102.62
EKVX	99.27
HOP-62	95.37
HOP-92	97.81
NCI-H226	83.18
NCI-H23	94.28
NCI-H460	98.68
NCI-H522	91.10
Colon Cancer	
HCC-2998	102.36
HCT-116	92.72
HCT-15	103.38
HT29	97.64
SW-620	99.71
CNS Cancer	
SF-268	98.12
SF-295	105.55
SF-539	106.06
SNB-19	104.86
SNB-75	116.58
U251	109.29
Melanoma	
LOX IMVI	92.36
MALME-3M	112.01
M14	103.21
MDA-MB-435	112.19
SK-MEL-2	97.15
SK-MEL-28	123.54
SK-MEL-5	92.89
UACC-257	103.31
UACC-62	114.54
Ovarian Cancer	
IGROV1	96.39
OVCAR-3	91.36
OVCAR-4	102.00
OVCAR-5	109.51
OVCAR-8	93.71
NCI/ADR-RES	97.58
SK-OV-3	102.42
Renal Cancer	
A498	93.81
ACHN	95.57
CAKI-1	101.19
RXF 393	108.19
SN12C	101.64
TK-10	129.98
UO-31	66.48
Prostate Cancer	
PC-3	90.58
DU-145	90.66
Breast Cancer	
MCF7	87.11
MDA-MB-231/ATCC	86.79
HS 578T	118.00
BT-549	93.10
T-47D	95.65
MDA-MB-468	96.86
Mean	99.92
Delta	33.44
Range	63.50

Mean Growth Percent - Growth Percent

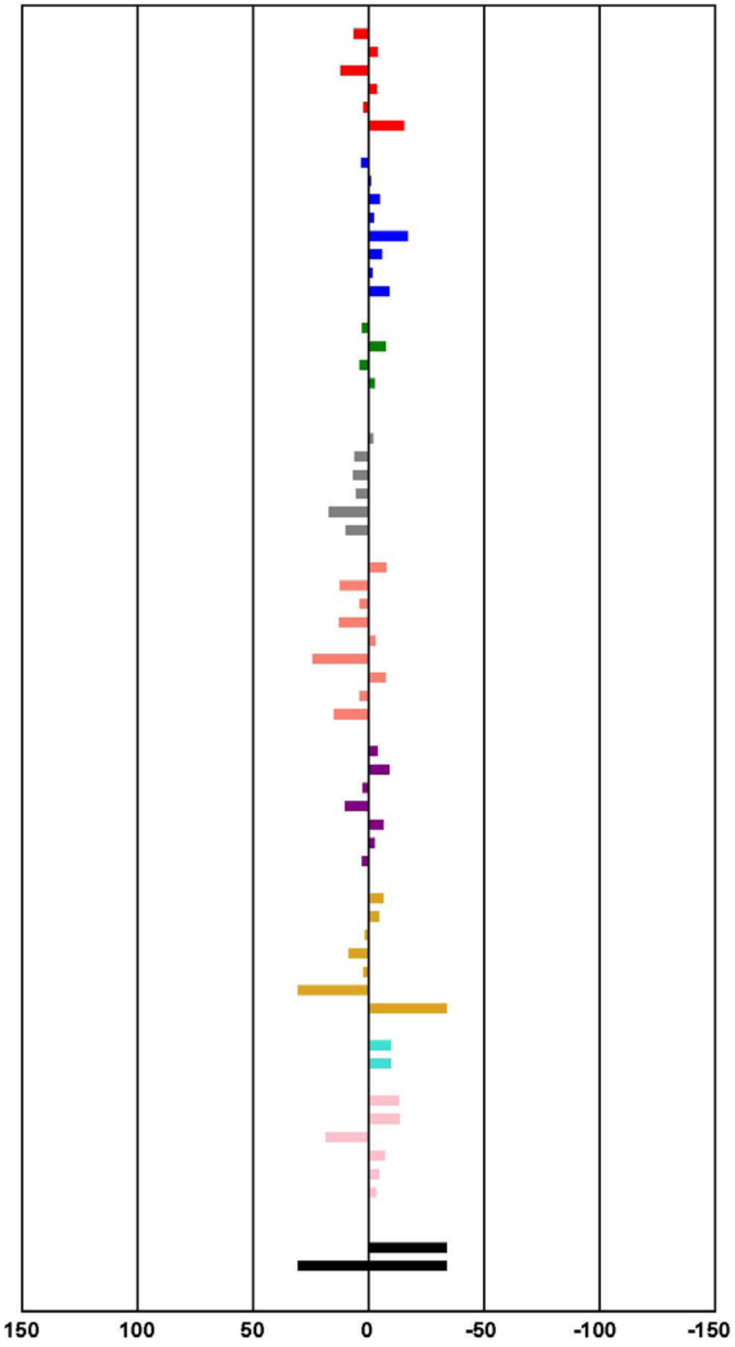

Figure S6: One dose assay (10µM) NCI 60 cell lines growth data for compound 11

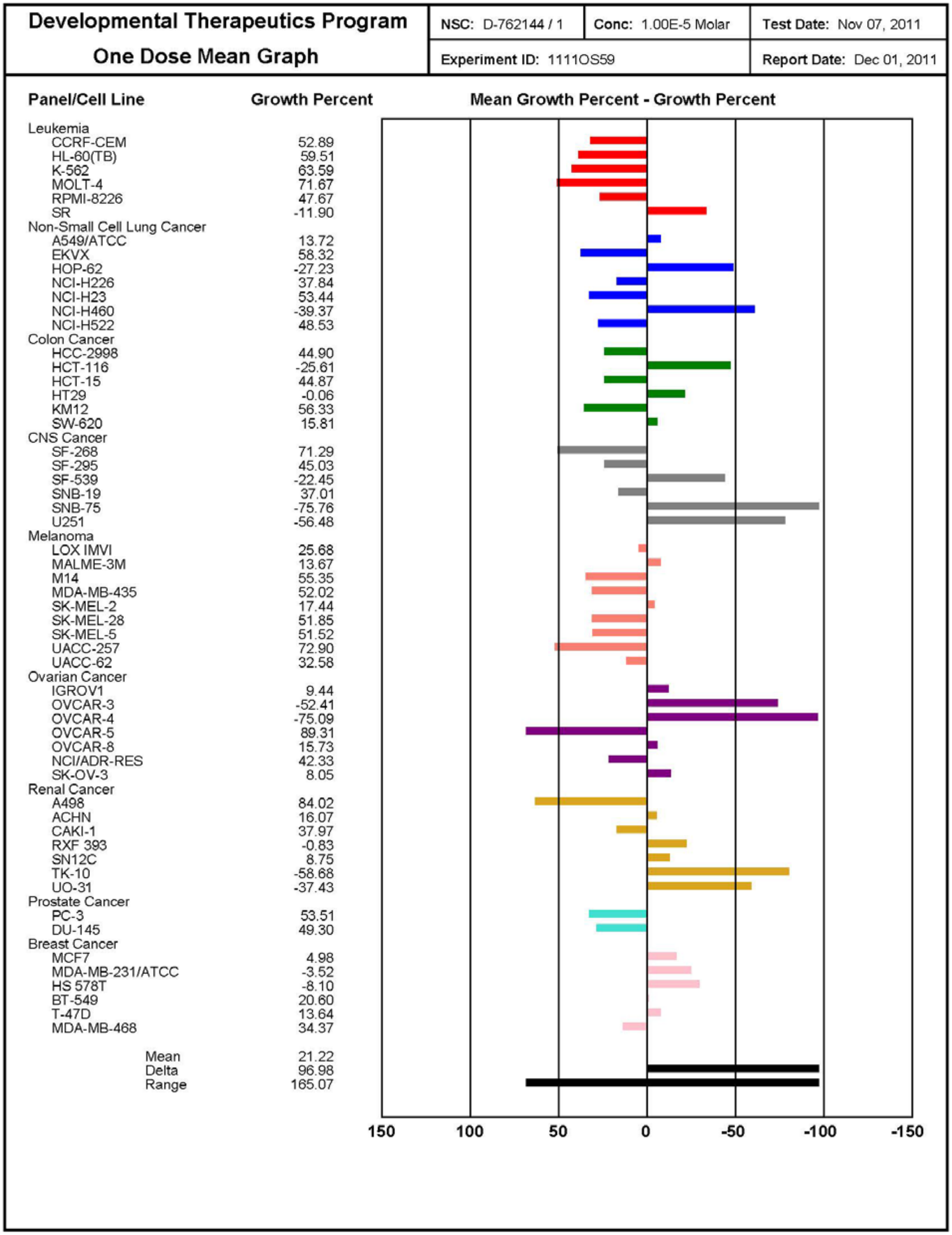

Figure S7: One dose assay (10μM) NCI 60 cell lines growth data for compound 13

Developmental Therapeutics Program One Dose Mean Graph	NSC: D-762123 / 1	Conc: 1.00E-5 Molar	Test Date: Nov 07, 2011
	Experiment ID: 1111OS59		Report Date: Dec 01, 2011

Panel/Cell Line	Growth Percent
**Leukemia**	
CCRF-CEM	30.14
HL-60(TB)	25.56
K-562	10.38
MOLT-4	0.60
RPMI-8226	23.70
SR	-1.72
**Non-Small Cell Lung Cancer**	
A549/ATCC	2.33
EKVX	39.21
HOP-62	18.38
NCI-H226	18.95
NCI-H23	22.98
NCI-H460	-22.58
NCI-H522	0.45
**Colon Cancer**	
HCC-2998	31.50
HCT-116	4.15
HCT-15	18.00
HT29	2.15
SW-620	13.82
**CNS Cancer**	
SF-268	29.98
SF-295	14.68
SF-539	15.52
SNB-19	29.17
SNB-75	47.07
U251	15.61
**Melanoma**	
LOX IMVI	9.65
MALME-3M	28.24
M14	16.59
MDA-MB-435	13.44
SK-MEL-2	-11.32
SK-MEL-28	29.95
SK-MEL-5	17.02
UACC-257	10.61
UACC-62	22.48
**Ovarian Cancer**	
IGROV1	21.18
OVCAR-3	30.58
OVCAR-4	23.68
OVCAR-5	34.32
OVCAR-8	9.99
NCI/ADR-RES	17.92
SK-OV-3	28.00
**Renal Cancer**	
A498	3.82
ACHN	9.55
CAKI-1	29.02
RXF 393	-0.13
SN12C	21.08
TK-10	4.35
UO-31	15.65
**Prostate Cancer**	
PC-3	23.21
DU-145	19.71
**Breast Cancer**	
MCF7	2.13
MDA-MB-231/ATCC	26.11
HS 578T	43.42
BT-549	54.44
T-47D	23.89
MDA-MB-468	11.94
Mean	17.83
Delta	40.41
Range	77.02

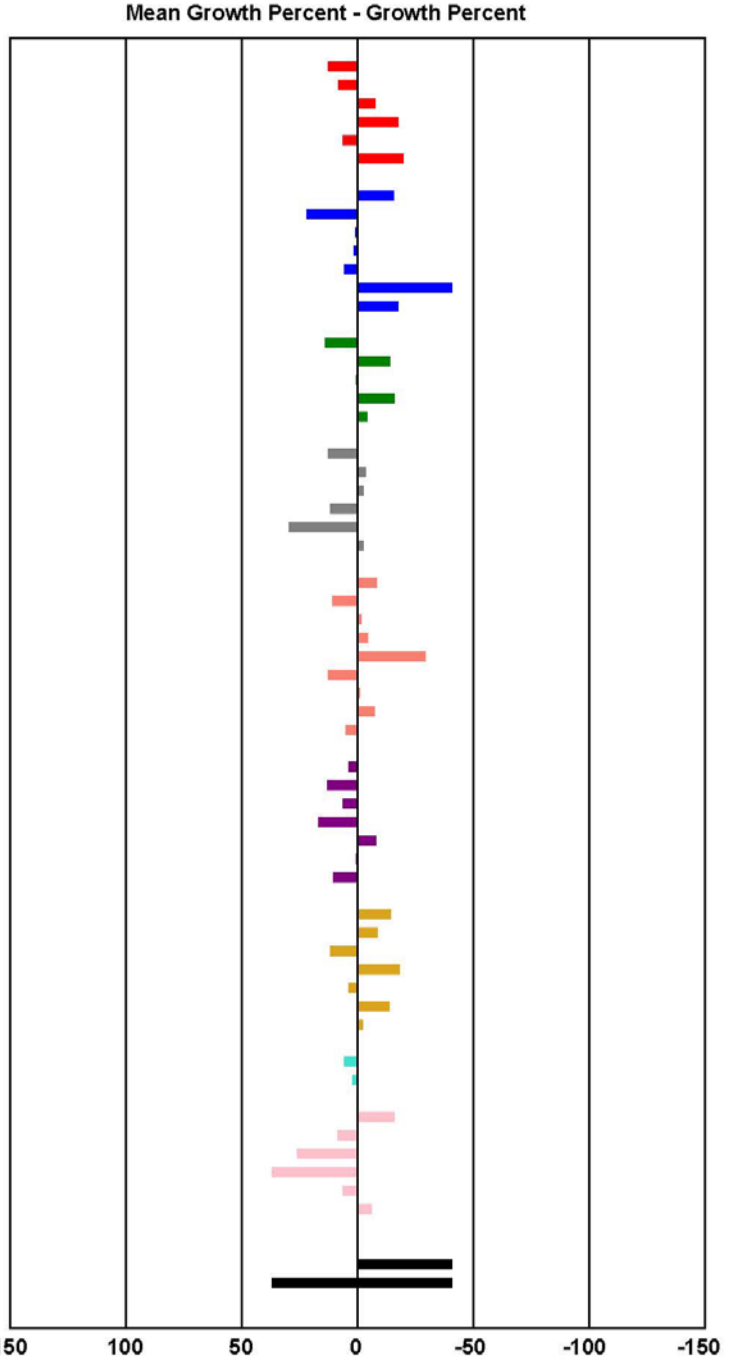

Figure S8: One dose assay (10µM) NCI 60 cell lines growth data for compound 15

Developmental Therapeutics Program	NSC: D-762141 / 1	Conc: 1.00E-5 Molar	Test Date: Nov 07, 2011
One Dose Mean Graph	Experiment ID: 1111OS59		Report Date: Dec 01, 2011

Panel/Cell Line	Growth Percent
**Leukemia**	
CCRF-CEM	100.24
HL-60(TB)	96.08
K-562	103.11
MOLT-4	94.31
RPMI-8226	97.57
SR	88.11
**Non-Small Cell Lung Cancer**	
A549/ATCC	91.87
EKVX	92.96
HOP-62	85.85
HOP-92	90.52
NCI-H226	87.99
NCI-H23	80.13
NCI-H460	97.59
NCI-H522	79.39
**Colon Cancer**	
HCC-2998	99.45
HCT-116	93.01
HCT-15	104.76
HT29	97.79
SW-620	93.49
**CNS Cancer**	
SF-268	104.29
SF-295	92.48
SF-539	112.67
SNB-19	105.72
SNB-75	104.51
U251	88.15
**Melanoma**	
LOX IMVI	78.63
MALME-3M	104.97
M14	100.82
MDA-MB-435	107.31
SK-MEL-2	80.54
SK-MEL-28	108.54
SK-MEL-5	79.30
UACC-257	97.01
UACC-62	106.87
**Ovarian Cancer**	
IGROV1	110.90
OVCAR-3	103.37
OVCAR-4	105.24
OVCAR-5	110.60
OVCAR-8	84.12
NCI/ADR-RES	90.17
SK-OV-3	103.79
**Renal Cancer**	
A498	88.01
ACHN	101.02
CAKI-1	101.06
RXF 393	93.71
SN12C	94.20
TK-10	119.84
UO-31	63.76
**Prostate Cancer**	
PC-3	82.32
DU-145	120.03
**Breast Cancer**	
MCF7	81.17
MDA-MB-231/ATCC	72.64
HS 578T	116.04
BT-549	89.71
T-47D	86.02
MDA-MB-468	87.89
Mean	95.56
Delta	31.80
Range	56.27

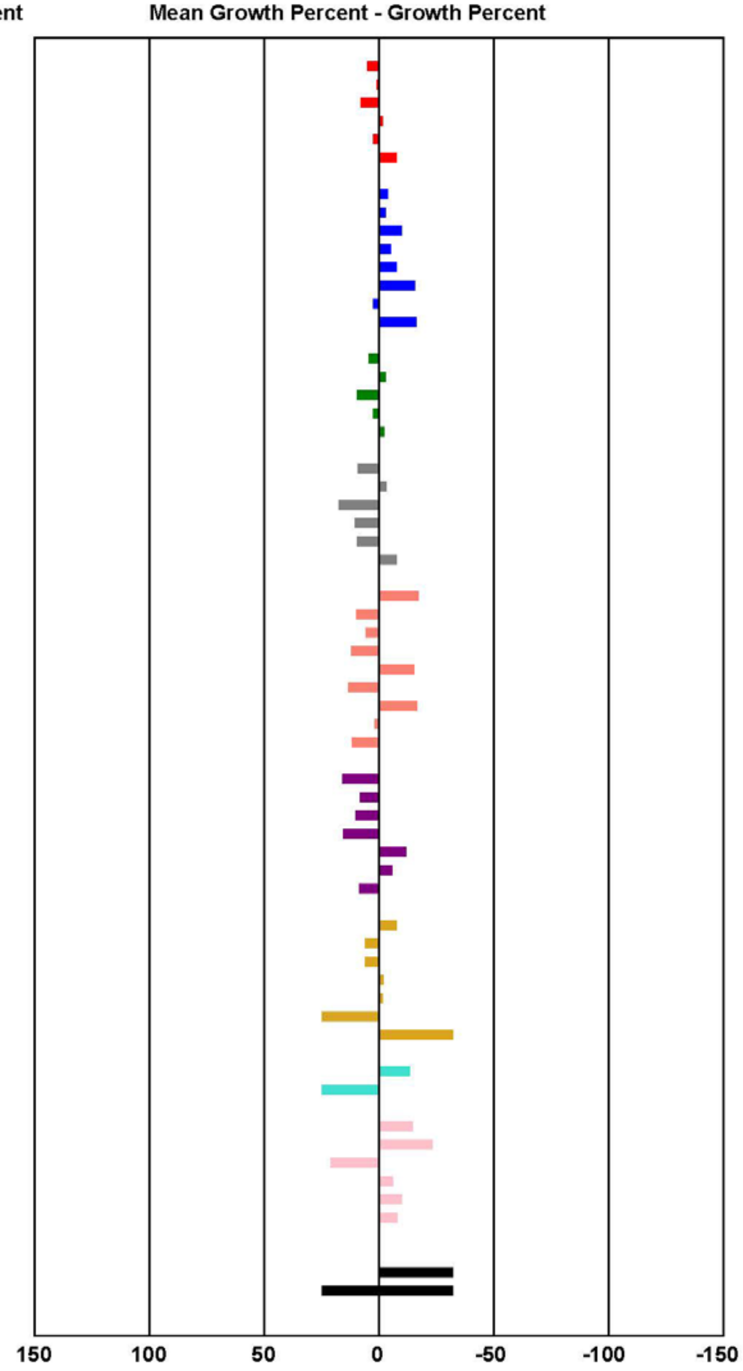

Figure S9: One dose assay (10µM) NCI 60 cell lines growth data for compound 16

Developmental Therapeutics Program One Dose Mean Graph	NSC: D-762142 / 1	Conc: 1.00E-5 Molar	Test Date: Nov 07, 2011
	Experiment ID: 1111OS59		Report Date: Dec 01, 2011

Panel/Cell Line	Growth Percent
**Leukemia**	
CCRF-CEM	113.06
HL-60(TB)	95.29
K-562	115.84
MOLT-4	101.55
RPMI-8226	107.39
SR	104.21
**Non-Small Cell Lung Cancer**	
A549/ATCC	115.04
EKVX	106.32
HOP-62	93.00
NCI-H226	100.51
NCI-H23	100.34
NCI-H460	117.51
NCI-H522	96.62
**Colon Cancer**	
HCC-2998	108.88
HCT-116	99.62
HCT-15	106.82
HT29	109.73
KM12	106.56
SW-620	106.94
**CNS Cancer**	
SF-268	108.21
SF-295	101.84
SF-539	122.08
SNB-19	96.36
SNB-75	74.02
U251	123.71
**Melanoma**	
LOX IMVI	92.42
MALME-3M	114.45
M14	117.63
MDA-MB-435	117.55
SK-MEL-28	111.90
SK-MEL-5	101.93
UACC-257	141.83
UACC-62	105.25
**Ovarian Cancer**	
IGROV1	103.93
OVCAR-3	107.05
OVCAR-4	96.05
OVCAR-5	113.69
OVCAR-8	109.17
NCI/ADR-RES	103.15
SK-OV-3	111.11
**Renal Cancer**	
A498	128.72
ACHN	111.51
CAKI-1	100.88
RXF 393	112.56
SN12C	98.42
TK-10	135.74
UO-31	76.22
**Prostate Cancer**	
PC-3	100.01
DU-145	116.08
**Breast Cancer**	
MCF7	80.53
MDA-MB-231/ATCC	77.64
HS 578T	153.39
BT-549	94.73
T-47D	76.47
MDA-MB-468	98.95
Mean	106.19
Delta	32.17
Range	79.37

Mean Growth Percent - Growth Percent

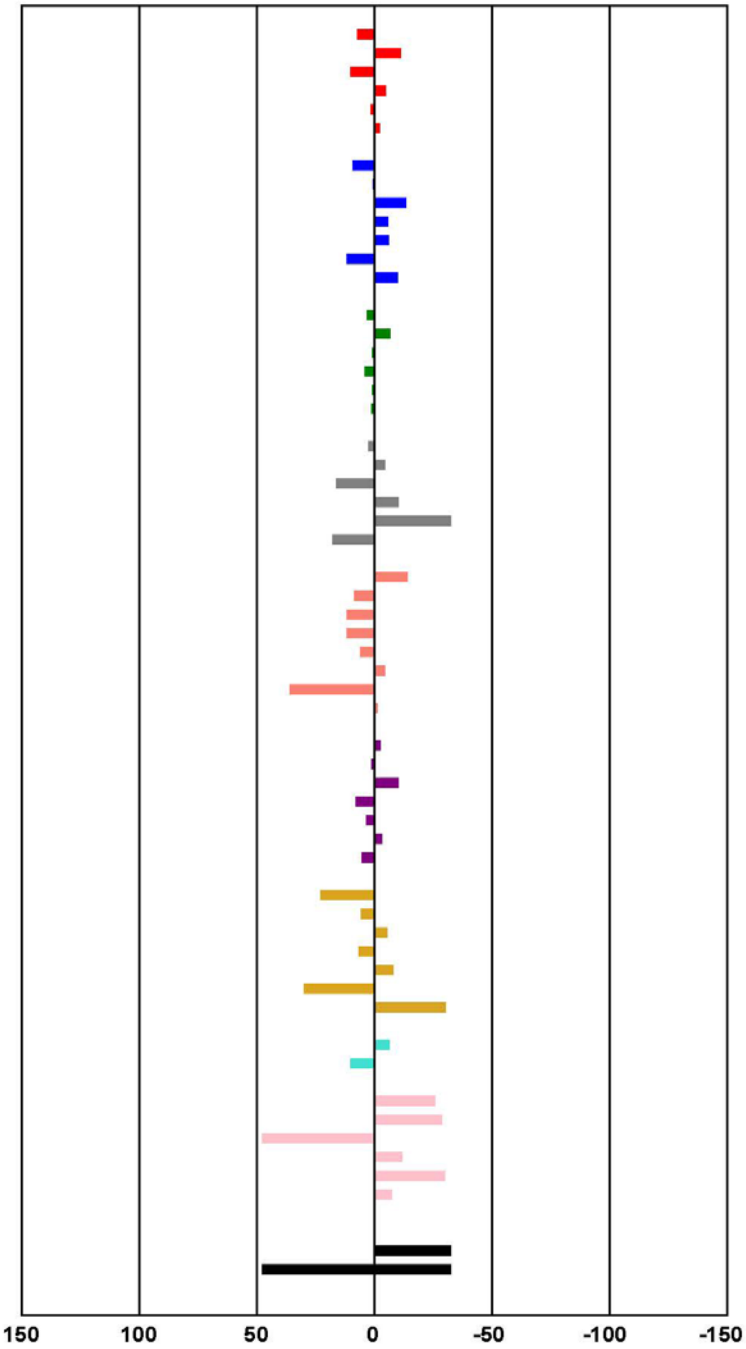

Figure S10: One dose assay (10μM) NCI 60 cell lines growth data for compound 17

Developmental Therapeutics Program	NSC: D-762143 / 1	Conc: 1.00E-5 Molar	Test Date: Nov 07, 2011
One Dose Mean Graph	Experiment ID: 1111OS59		Report Date: Dec 01, 2011

Panel/Cell Line	Growth Percent
Leukemia	
CCRF-CEM	106.20
HL-60(TB)	96.23
K-562	105.15
MOLT-4	96.31
RPMI-8226	94.35
SR	97.18
Non-Small Cell Lung Cancer	
A549/ATCC	98.58
EKVX	99.50
HOP-62	94.70
NCI-H226	91.70
NCI-H23	93.08
NCI-H460	105.29
NCI-H522	99.47
Colon Cancer	
HCC-2998	102.35
HCT-116	100.17
HCT-15	119.98
HT29	103.77
KM12	107.85
SW-620	102.81
CNS Cancer	
SF-268	112.74
SF-295	91.36
SF-539	112.66
SNB-19	108.67
SNB-75	78.18
U251	107.00
Melanoma	
LOX IMVI	87.87
MALME-3M	128.81
M14	108.16
MDA-MB-435	108.44
SK-MEL-2	122.87
SK-MEL-28	111.84
SK-MEL-5	94.35
UACC-257	101.70
UACC-62	115.32
Ovarian Cancer	
IGROV1	112.42
OVCAR-3	109.03
OVCAR-4	98.73
OVCAR-5	111.60
OVCAR-8	99.53
NCI/ADR-RES	94.18
SK-OV-3	110.11
Renal Cancer	
A498	102.74
ACHN	106.23
CAKI-1	92.74
RXF 393	98.52
SN12C	93.57
TK-10	144.84
UO-31	67.06
Prostate Cancer	
PC-3	91.57
DU-145	112.95
Breast Cancer	
MCF7	75.50
MDA-MB-231/ATCC	68.25
HS 578T	120.83
BT-549	94.35
T-47D	86.83
MDA-MB-468	99.98
Mean	101.72
Delta	34.66
Range	77.78

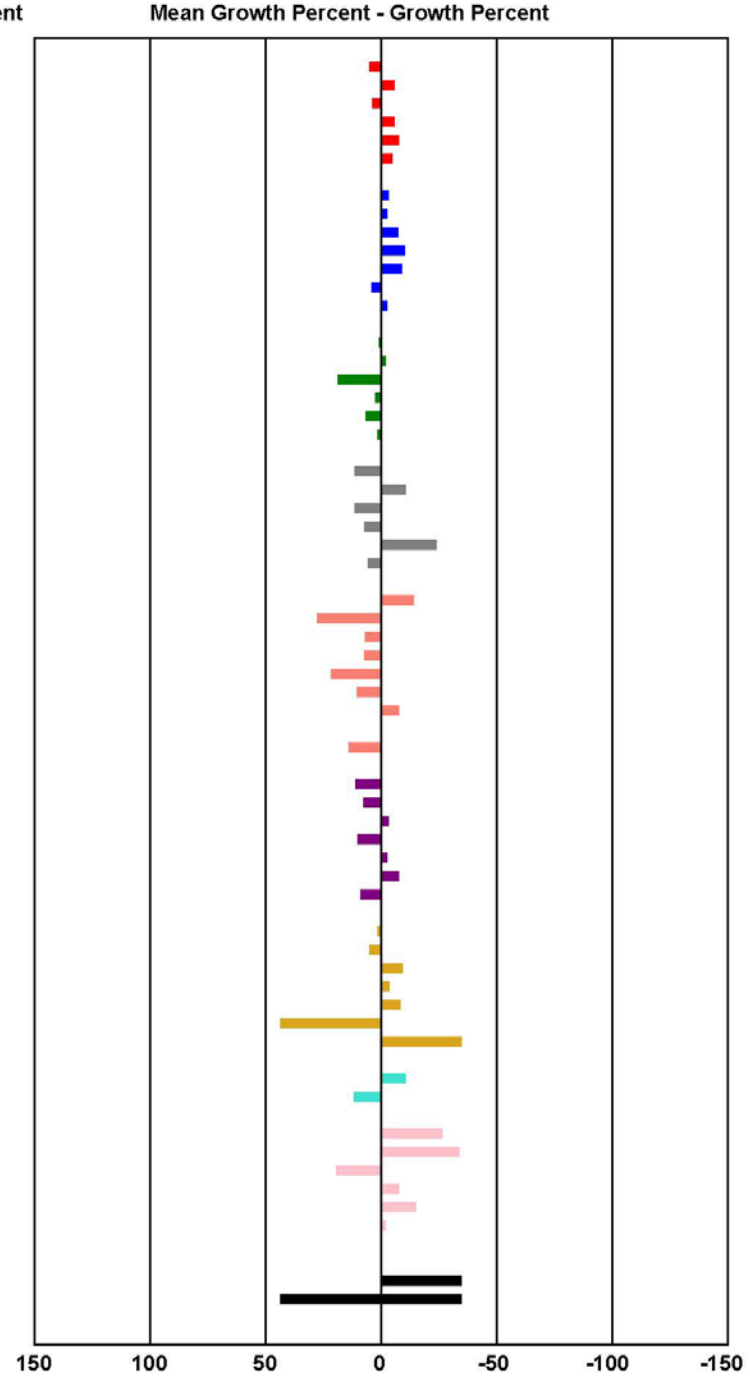

Figure S11: Five Dose Assay data graph from NCI 60 cell lines growth for compound 11

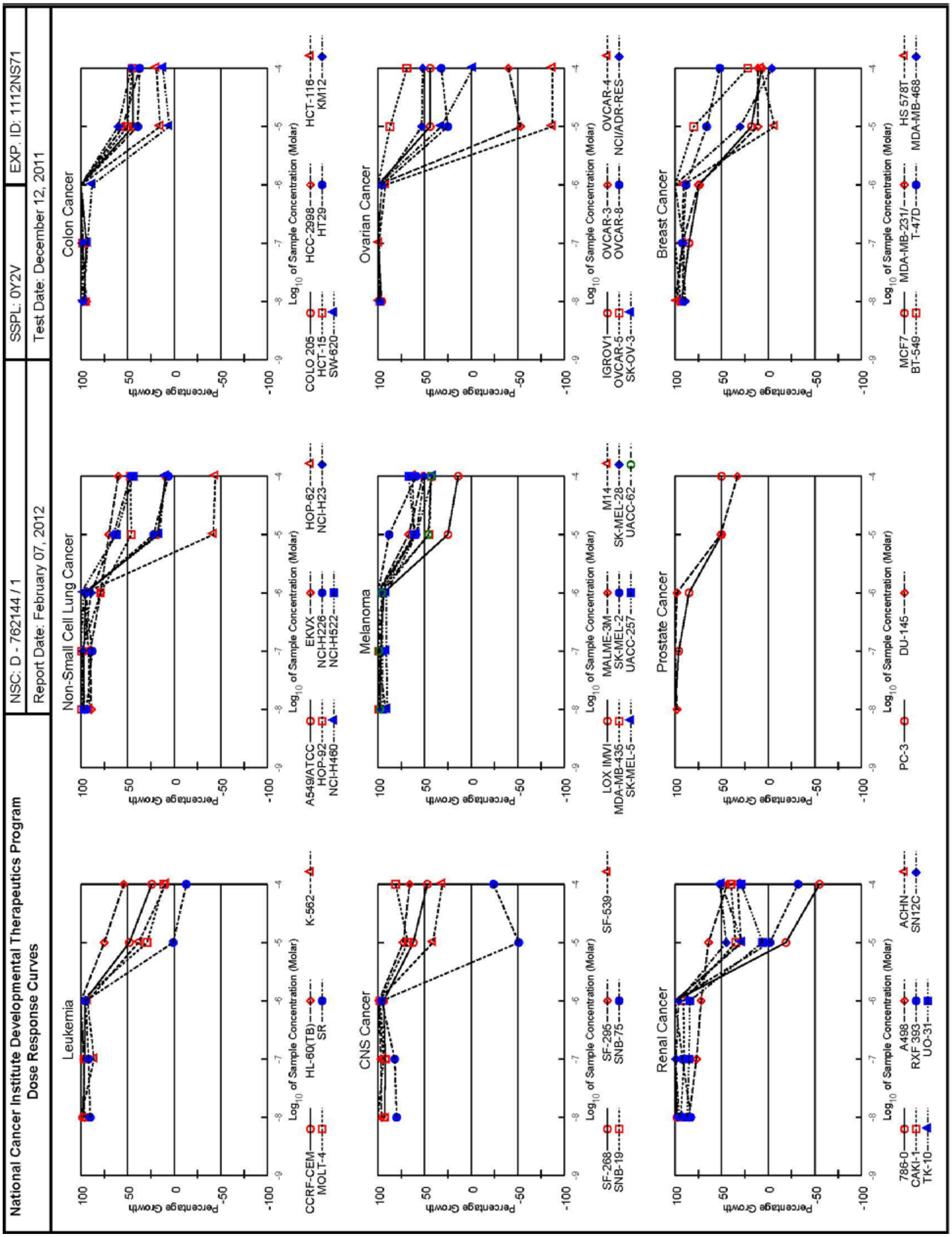

Figure S12: Complete Five Dose Assay data from NCI 60 cell lines for compound 11

National Cancer Institute Developmental Therapeutics Program
In-Vitro Testing Results

NSC : D - 762144 / 1	Experiment ID : 1112NS71	Test Type : 08	Units : Molar
Report Date : February 07, 2012	Test Date : December 12, 2011	QNS :	MC :
COMI : CM-5-278-4-2 (111980)	Stain Reagent : SRB Dual-Pass Related	SSPL : 0Y2V	

			Log10 Concentration												
	Time		Mean Optical Densities					Percent Growth							
Panel/Cell Line	Zero	Ctrl	-8.0	-7.0	-6.0	-5.0	-4.0	-8.0	-7.0	-6.0	-5.0	-4.0	GI50	TGI	LC50
**Leukemia**															
CCRF-CEM	0.438	2.023	1.985	1.958	1.961	1.199	0.811	98	96	96	48	24	9.08E-6	> 1.00E-4	> 1.00E-4
HL-60(TB)	0.860	2.718	2.644	2.667	2.942	2.257	1.864	96	97	112	75	54	> 1.00E-4	> 1.00E-4	> 1.00E-4
K-562	0.167	1.357	1.415	1.192	1.277	0.621	0.280	105	86	93	38	9	6.10E-6	> 1.00E-4	> 1.00E-4
MOLT-4	0.461	1.988	1.945	1.936	2.078	0.910	0.635	97	97	106	29	11	5.38E-6	> 1.00E-4	> 1.00E-4
SR	0.316	1.902	1.744	1.770	1.825	0.337	0.276	90	92	95	1	-13	3.03E-6	1.24E-5	> 1.00E-4
**Non-Small Cell Lung Cancer**															
A549/ATCC	0.314	1.552	1.513	1.517	1.494	0.533	0.420	97	97	95	18	9	3.83E-6	> 1.00E-4	> 1.00E-4
EKVX	0.576	1.271	1.196	1.199	1.123	1.066	0.991	89	90	79	70	60	> 1.00E-4	> 1.00E-4	> 1.00E-4
HOP-62	0.303	0.745	0.705	0.708	0.727	0.175	0.169	91	92	96	-42	-44	2.15E-6	4.94E-6	> 1.00E-4
HOP-92	0.988	1.431	1.426	1.428	1.338	1.191	1.195	99	99	79	46	47	7.49E-6	> 1.00E-4	> 1.00E-4
NCI-H226	0.638	1.286	1.248	1.207	1.257	0.784	0.678	94	88	95	22	6	4.19E-6	> 1.00E-4	> 1.00E-4
NCI-H23	0.861	2.362	2.310	2.327	2.203	1.843	1.564	96	98	89	65	47	6.75E-5	> 1.00E-4	> 1.00E-4
NCI-H460	0.285	2.455	2.519	2.548	2.382	0.622	0.470	103	104	97	16	9	3.76E-6	> 1.00E-4	> 1.00E-4
NCI-H522	0.678	1.462	1.439	1.460	1.505	1.162	1.024	97	100	105	62	44	4.64E-5	> 1.00E-4	> 1.00E-4
**Colon Cancer**															
COLO 205	0.279	0.969	0.949	0.931	0.997	0.580	0.585	97	94	104	44	44	7.84E-6	> 1.00E-4	> 1.00E-4
HCC-2998	0.591	1.723	1.654	1.715	1.756	1.216	1.010	94	99	103	55	37	1.93E-5	> 1.00E-4	> 1.00E-4
HCT-116	0.219	1.617	1.624	1.651	1.646	0.422	0.486	100	102	102	15	19	3.93E-6	> 1.00E-4	> 1.00E-4
HCT-15	0.292	1.980	1.896	1.947	2.048	1.133	1.055	95	98	104	50	45	9.91E-6	> 1.00E-4	> 1.00E-4
HT29	0.198	1.156	1.200	1.222	1.235	0.570	0.551	105	107	108	39	37	6.90E-6	> 1.00E-4	> 1.00E-4
KM12	0.493	2.113	2.077	2.081	2.115	1.458	1.239	98	98	100	60	46	5.12E-5	> 1.00E-4	> 1.00E-4
SW-620	0.244	1.693	1.654	1.590	1.518	0.313	0.420	97	93	88	5	12	2.86E-6	> 1.00E-4	> 1.00E-4
**CNS Cancer**															
SF-268	0.496	1.653	1.577	1.564	1.585	1.218	1.046	93	92	94	62	47	6.78E-5	> 1.00E-4	> 1.00E-4
SF-295	0.819	2.588	2.521	2.535	2.566	2.116	1.979	96	97	99	73	66	> 1.00E-4	> 1.00E-4	> 1.00E-4
SF-539	0.695	1.786	1.786	1.715	1.814	1.144	1.039	100	94	103	41	31	7.17E-6	> 1.00E-4	> 1.00E-4
SNB-19	0.650	1.915	1.826	1.817	1.888	1.509	1.673	93	92	98	68	81	> 1.00E-4	> 1.00E-4	> 1.00E-4
SNB-75	0.735	1.242	1.141	1.150	1.221	0.358	0.560	80	82	96	-51	-24	2.05E-6	4.48E-6	.
**Melanoma**															
LOX IMVI	0.255	1.633	1.612	1.590	1.551	0.606	0.445	98	97	94	25	14	4.38E-6	> 1.00E-4	> 1.00E-4
MALME-3M	0.588	1.774	1.772	1.842	1.786	1.383	1.201	100	106	101	67	52	> 1.00E-4	> 1.00E-4	> 1.00E-4
M14	0.415	1.396	1.319	1.447	1.450	1.016	1.038	92	105	105	61	63	> 1.00E-4	> 1.00E-4	> 1.00E-4
MDA-MB-435	0.473	1.999	1.979	1.982	2.027	1.153	1.132	99	99	102	45	43	8.02E-6	> 1.00E-4	> 1.00E-4
SK-MEL-2	0.861	1.768	1.789	1.824	1.850	1.663	1.395	102	106	109	88	59	> 1.00E-4	> 1.00E-4	> 1.00E-4
SK-MEL-28	0.507	1.332	1.309	1.377	1.334	0.994	0.922	97	106	100	59	50	> 1.00E-4	> 1.00E-4	> 1.00E-4
SK-MEL-5	0.523	2.400	2.220	2.241	2.238	1.627	1.286	90	91	91	59	41	3.05E-5	> 1.00E-4	> 1.00E-4
UACC-257	0.550	1.294	1.257	1.258	1.267	1.002	1.049	95	95	96	61	67	> 1.00E-4	> 1.00E-4	> 1.00E-4
UACC-62	0.579	2.143	2.119	2.120	2.080	1.306	1.250	98	99	96	46	43	8.49E-6	> 1.00E-4	> 1.00E-4
**Ovarian Cancer**															
IGROV1	0.590	1.333	1.305	1.335	1.368	0.920	0.914	96	100	105	44	44	8.07E-6	> 1.00E-4	> 1.00E-4
OVCAR-3	0.517	1.343	1.351	1.386	1.422	0.245	0.310	101	105	110	-53	-40	2.33E-6	4.74E-6	.
OVCAR-4	0.534	1.091	1.086	1.083	1.045	0.068	0.070	99	99	92	-87	-87	1.71E-6	3.25E-6	6.19E-6
OVCAR-5	0.597	1.539	1.517	1.584	1.610	1.417	1.246	98	105	108	87	69	> 1.00E-4	> 1.00E-4	> 1.00E-4
OVCAR-8	0.417	1.796	1.764	1.809	1.739	0.757	0.856	98	101	96	25	32	4.41E-6	> 1.00E-4	> 1.00E-4
NCI/ADR-RES	0.532	1.782	1.807	1.792	1.738	1.195	1.184	102	101	96	53	52	> 1.00E-4	> 1.00E-4	> 1.00E-4
SK-OV-3	0.487	1.113	1.116	1.123	1.130	0.690	0.476	100	102	103	32	-2	5.62E-6	8.55E-5	> 1.00E-4
**Renal Cancer**															
786-0	0.834	2.295	2.263	2.322	2.378	0.673	0.375	98	102	106	-19	-55	2.79E-6	7.00E-6	7.20E-5
A498	1.307	1.779	1.709	1.670	1.647	1.611	1.515	85	77	72	64	44	5.05E-5	> 1.00E-4	> 1.00E-4
ACHN	0.312	1.332	1.385	1.380	1.442	0.606	0.646	105	105	111	29	33	5.51E-6	> 1.00E-4	> 1.00E-4
CAKI-1	0.638	1.954	1.880	1.820	1.835	1.099	1.166	94	90	91	35	40	5.39E-6	> 1.00E-4	> 1.00E-4
RXF 393	0.656	1.103	1.027	1.069	1.123	0.646	0.445	83	92	105	-2	-32	3.27E-6	9.66E-6	> 1.00E-4
SN12C	0.466	1.951	1.906	1.936	1.889	1.127	1.240	97	99	96	45	52	.	> 1.00E-4	> 1.00E-4
TK-10	0.738	1.336	1.308	1.340	1.524	0.906	1.039	95	101	131	28	50	.	> 1.00E-4	> 1.00E-4
UO-31	0.555	1.769	1.606	1.590	1.577	0.625	0.911	87	85	84	6	29	2.73E-6	> 1.00E-4	> 1.00E-4
**Prostate Cancer**															
PC-3	0.513	1.957	1.962	1.896	1.746	1.230	1.231	100	96	85	50	50	9.76E-6	> 1.00E-4	> 1.00E-4
DU-145	0.381	1.272	1.255	1.301	1.257	0.834	0.675	98	103	98	51	33	1.12E-5	> 1.00E-4	> 1.00E-4
**Breast Cancer**															
MCF7	0.268	1.547	1.452	1.355	1.219	0.499	0.361	93	85	74	18	7	2.71E-6	> 1.00E-4	> 1.00E-4
MDA-MB-231/ATCC	0.490	1.150	1.171	1.097	0.983	0.560	0.561	103	92	75	11	11	2.43E-6	> 1.00E-4	> 1.00E-4
HS 578T	1.204	1.930	1.914	1.869	1.863	1.119	1.261	98	92	91	-7	8	2.61E-6	.	> 1.00E-4
BT-549	0.840	1.463	1.427	1.528	1.611	1.337	0.980	94	110	124	80	22	3.31E-5	> 1.00E-4	> 1.00E-4
T-47D	0.624	1.623	1.536	1.540	1.503	1.288	1.148	91	92	88	66	52	> 1.00E-4	> 1.00E-4	> 1.00E-4
MDA-MB-468	0.549	0.844	0.811	0.816	0.888	0.638	0.526	89	91	115	30	-4	5.82E-6	7.54E-5	> 1.00E-4

Figure S13: Complete Five Dose Assay data from NCI 60 cell lines for compound 11

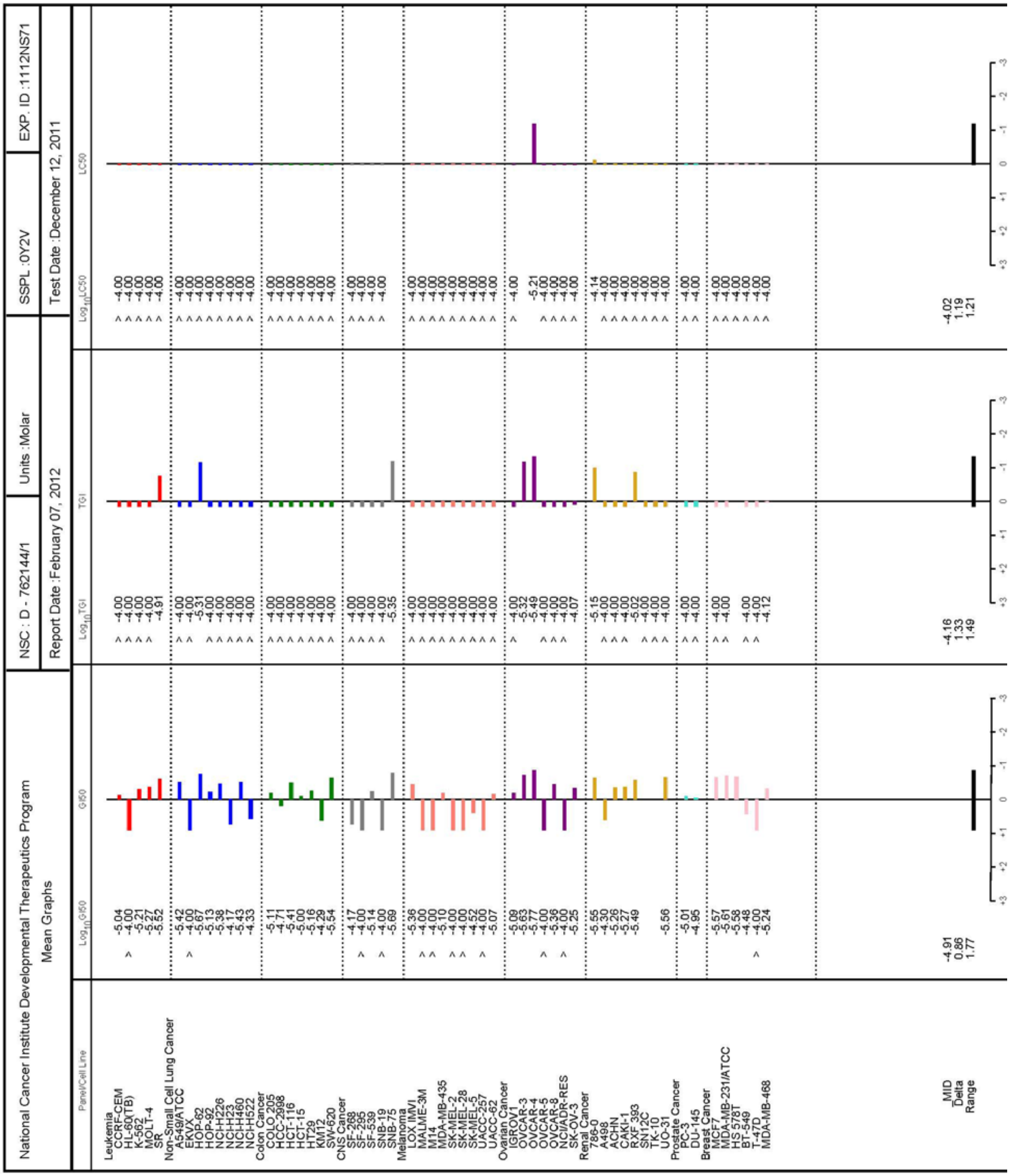

Figure S14: Five Dose Assay data graph from NCI 60 cell lines for compound 13

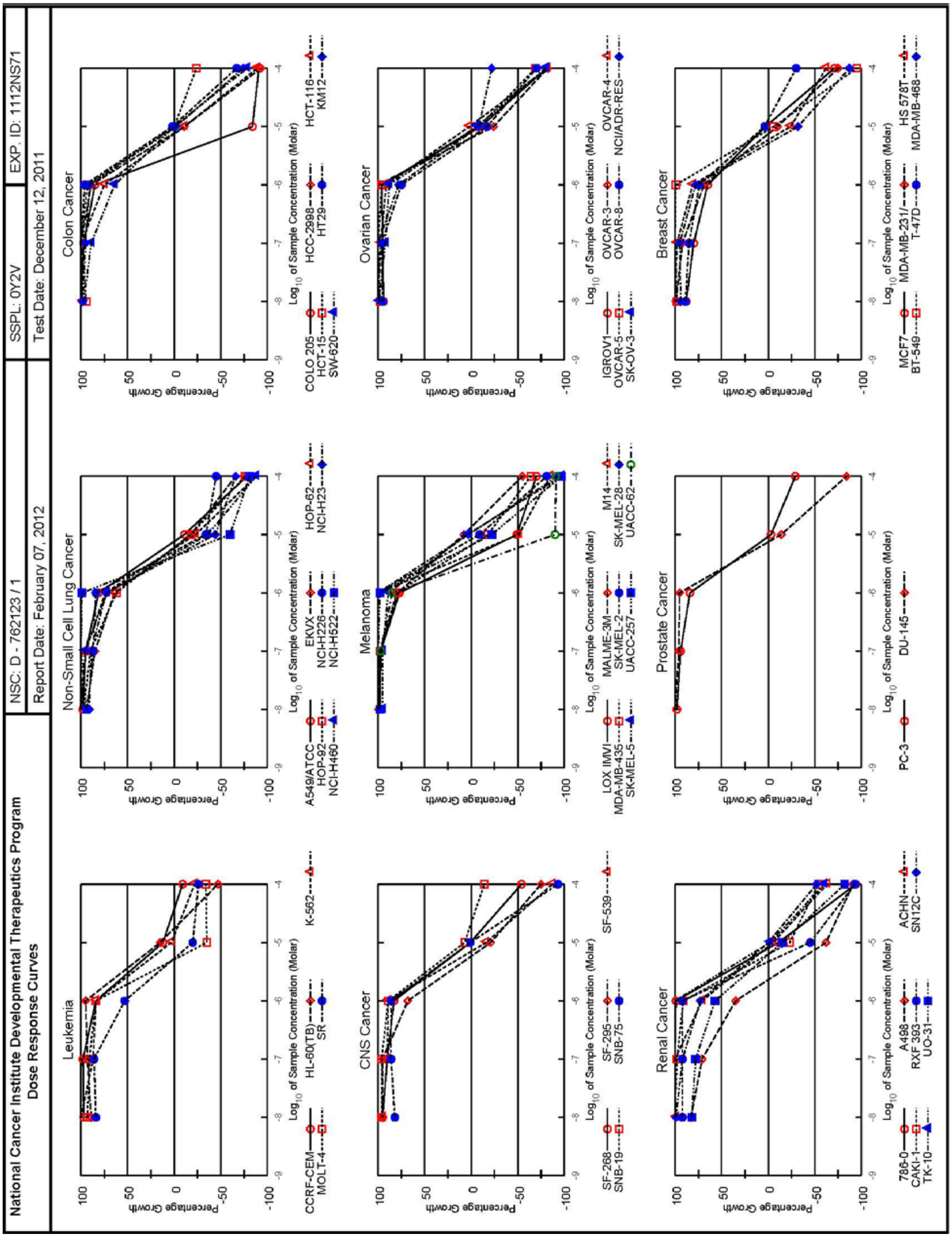

Figure S15: Complete Five Dose Assay data from NCI 60 cell lines for compound 13

National Cancer Institute Developmental Therapeutics Program
In-Vitro Testing Results

NSC : D - 762123 / 1	Experiment ID : 1112NS71	Test Type : 08	Units : Molar
Report Date : February 07, 2012	Test Date : December 12, 2011	QNS :	MC :
COMI : CM3-112-5 (111402)	Stain Reagent : SRB Dual-Pass Related	SSPL : 0Y2V	

			Log10 Concentration												
	Time		Mean Optical Densities					Percent Growth							
Panel/Cell Line	Zero	Ctrl	-8.0	-7.0	-6.0	-5.0	-4.0	-8.0	-7.0	-6.0	-5.0	-4.0	GI50	TGI	LC50
**Leukemia**															
CCRF-CEM	0.438	2.083	2.030	2.052	1.821	0.643	0.398	97	98	84	12	-9	2.99E-6	3.74E-5	> 1.00E-4
HL-60(TB)	0.860	2.628	2.434	2.502	2.537	1.121	0.460	89	93	95	15	-47	3.63E-6	1.74E-5	> 1.00E-4
K-562	0.167	1.390	1.401	1.230	1.193	0.200	0.131	101	87	84	3	-22	2.61E-6	1.28E-5	> 1.00E-4
MOLT-4	0.461	1.879	1.785	1.752	1.652	0.300	0.306	93	91	84	-35	-34	1.93E-6	5.08E-6	> 1.00E-4
SR	0.316	1.791	1.549	1.585	1.105	0.254	0.235	84	86	53	-20	-26	1.12E-6	5.37E-6	> 1.00E-4
**Non-Small Cell Lung Cancer**															
A549/ATCC	0.314	1.418	1.392	1.366	1.207	0.281	0.071	98	95	81	-11	-78	2.17E-6	7.65E-6	3.87E-5
EKVX	0.576	1.239	1.201	1.142	1.000	0.471	0.197	94	85	64	-18	-66	1.48E-6	5.99E-6	4.64E-5
HOP-62	0.303	0.691	0.709	0.670	0.591	0.233	0.037	105	95	74	-23	-88	1.77E-6	5.77E-6	2.60E-5
HOP-92	0.988	1.386	1.374	1.347	1.237	0.815	0.239	97	90	62	-18	-76	1.43E-6	6.04E-6	3.60E-5
NCI-H226	0.638	1.272	1.226	1.191	1.168	0.422	0.353	93	87	84	-34	-45	1.93E-6	5.15E-6	> 1.00E-4
NCI-H23	0.861	2.235	2.117	2.081	1.860	0.482	0.291	91	89	73	-44	-66	1.57E-6	4.20E-6	1.86E-5
NCI-H460	0.285	2.290	2.302	2.215	1.736	0.182	0.035	101	96	72	-36	-88	1.61E-6	4.64E-6	1.85E-5
NCI-H522	0.678	1.392	1.347	1.390	1.383	0.274	0.126	94	100	99	-60	-81	2.03E-6	4.20E-6	8.70E-6
**Colon Cancer**															
COLO 205	0.279	0.955	0.963	0.935	0.851	0.045	0.024	101	97	85	-84	-91	1.60E-6	3.17E-6	6.28E-6
HCC-2998	0.591	1.600	1.593	1.673	1.492	0.527	0.040	99	107	89	-11	-93	2.47E-6	7.78E-6	2.98E-5
HCT-116	0.219	1.514	1.484	1.607	1.196	0.195	0.025	98	107	75	-11	-89	1.97E-6	7.47E-6	3.18E-5
HCT-15	0.292	1.807	1.723	1.803	1.700	0.291	0.222	94	100	93	.	-24	2.88E-6	9.92E-6	> 1.00E-4
HT29	0.198	1.114	1.112	1.135	1.081	0.216	0.065	100	102	96	2	-67	3.09E-6	1.07E-5	5.61E-5
KM12	0.493	2.032	2.003	1.962	1.891	0.484	0.130	98	95	91	-2	-74	2.76E-6	9.53E-6	4.67E-5
SW-620	0.244	1.548	1.511	1.407	1.076	0.247	0.054	97	89	64	.	-78	1.65E-6	1.01E-5	4.40E-5
**CNS Cancer**															
SF-268	0.496	1.531	1.483	1.423	1.358	0.506	0.228	95	90	83	1	-54	2.53E-6	1.04E-5	8.41E-5
SF-295	0.819	2.664	2.568	2.563	2.069	0.644	0.207	95	95	68	-21	-75	1.58E-6	5.75E-6	3.43E-5
SF-539	0.695	1.677	1.628	1.578	1.572	0.585	0.090	95	90	89	-16	-87	2.36E-6	7.07E-6	3.02E-5
SNB-19	0.650	1.873	1.829	1.825	1.743	0.737	0.557	96	96	89	7	-14	3.01E-6	2.15E-5	> 1.00E-4
SNB-75	0.735	1.285	1.188	1.209	1.206	0.742	0.049	82	86	86	1	-93	2.65E-6	1.03E-5	3.48E-5
**Melanoma**															
LOX IMVI	0.255	1.506	1.480	1.471	1.218	0.129	0.077	98	97	77	-49	-70	1.63E-6	4.06E-6	1.07E-5
MALME-3M	0.588	1.793	1.778	1.772	1.542	0.681	0.264	99	98	79	8	-55	2.56E-6	1.33E-5	8.29E-5
M14	0.415	1.237	1.254	1.329	1.286	0.202	0.063	102	111	106	-51	-85	2.27E-6	4.71E-6	9.79E-6
MDA-MB-435	0.473	1.925	1.902	1.903	1.634	0.387	0.172	98	98	80	-18	-64	2.02E-6	6.51E-6	5.00E-5
SK-MEL-2	0.861	1.601	1.603	1.669	1.671	0.780	0.166	100	109	109	-9	-81	3.16E-6	8.33E-6	3.71E-5
SK-MEL-28	0.507	1.416	1.437	1.487	1.457	0.534	0.033	102	108	105	3	-93	3.44E-6	1.07E-5	3.54E-5
SK-MEL-5	0.523	2.479	2.373	2.387	2.265	0.610	0.018	95	95	89	4	-97	2.90E-6	1.11E-5	3.46E-5
UACC-257	0.550	1.136	1.123	1.153	1.123	0.427	0.017	98	103	98	-22	-97	2.50E-6	6.51E-6	2.35E-5
UACC-62	0.579	1.948	1.981	1.913	1.738	0.059	0.051	102	97	85	-90	-91	1.58E-6	3.06E-6	5.91E-6
**Ovarian Cancer**															
IGROV1	0.590	1.284	1.245	1.251	1.255	0.532	0.112	94	95	96	-10	-81	2.71E-6	8.06E-6	3.66E-5
OVCAR-3	0.517	1.310	1.330	1.314	1.310	0.395	0.091	102	101	100	-24	-82	2.53E-6	6.43E-6	2.80E-5
OVCAR-4	0.534	1.141	1.140	1.123	1.088	0.544	0.097	100	97	91	2	-82	2.88E-6	1.04E-5	4.15E-5
OVCAR-5	0.597	1.453	1.436	1.539	1.422	0.551	0.186	98	110	96	-8	-69	2.78E-6	8.42E-6	4.91E-5
OVCAR-8	0.417	1.651	1.591	1.600	1.340	0.345	0.124	95	96	75	-17	-70	1.86E-6	6.48E-6	4.14E-5
NCI/ADR-RES	0.532	1.678	1.697	1.716	1.426	0.488	0.413	102	103	78	-8	-22	2.11E-6	8.00E-6	> 1.00E-4
SK-OV-3	0.487	1.113	1.109	1.062	1.037	0.459	0.100	99	92	88	-6	-80	2.54E-6	8.68E-6	3.98E-5
**Renal Cancer**															
786-0	0.834	2.220	2.217	2.229	2.203	0.746	0.053	100	101	99	-11	-94	2.79E-6	8.01E-6	2.98E-5
A498	1.307	1.744	1.669	1.620	1.460	0.494	0.114	83	71	35	-62	-91	3.86E-7	2.29E-6	7.48E-6
ACHN	0.312	1.277	1.264	1.286	0.999	0.297	0.117	99	101	71	-5	-63	1.90E-6	8.64E-6	6.07E-5
CAKI-1	0.638	1.856	1.882	1.832	1.743	0.492	0.292	102	98	91	-23	-54	2.28E-6	6.28E-6	7.32E-5
RXF 393	0.656	1.086	1.053	1.050	1.051	0.363	0.044	92	92	92	-45	-93	2.03E-6	4.70E-6	1.28E-5
SN12C	0.466	1.816	1.809	1.708	1.447	0.472	0.223	99	92	73	.	-52	2.06E-6	1.02E-5	9.10E-5
TK-10	0.738	1.246	1.259	1.334	1.447	0.721	0.298	102	117	139	-2	-60	4.27E-6	9.62E-6	6.79E-5
UO-31	0.555	1.719	1.508	1.464	1.217	0.474	0.103	82	78	57	-15	-82	1.25E-6	6.24E-6	3.38E-5
**Prostate Cancer**															
PC-3	0.513	1.913	1.891	1.826	1.684	0.496	0.363	98	94	84	-3	-29	2.43E-6	9.14E-6	> 1.00E-4
DU-145	0.381	1.201	1.222	1.166	1.157	0.328	0.060	103	96	95	-14	-84	2.58E-6	7.44E-6	3.26E-5
**Breast Cancer**															
MCF7	0.268	1.555	1.401	1.296	1.103	0.305	0.079	88	80	65	3	-71	1.74E-6	1.09E-5	5.23E-5
MDA-MB-231/ATCC	0.490	0.995	0.989	0.953	0.839	0.440	0.124	99	92	69	-10	-75	1.74E-6	7.44E-6	4.13E-5
HS 578T	1.204	1.793	1.791	1.780	1.679	0.917	0.457	100	98	81	-24	-62	1.96E-6	5.91E-6	4.84E-5
BT-549	0.840	1.393	1.384	1.470	1.386	0.793	0.044	98	114	99	-6	-95	2.93E-6	8.84E-6	3.15E-5
T-47D	0.624	1.643	1.536	1.486	1.381	0.665	0.440	89	85	74	4	-30	2.21E-6	1.31E-5	> 1.00E-4
MDA-MB-468	0.549	0.835	0.817	0.822	0.772	0.374	0.074	94	95	78	-32	-87	1.79E-6	5.12E-6	2.14E-5

Figure S16: Complete Five Dose Assay data from NCI 60 cell lines for compound 13

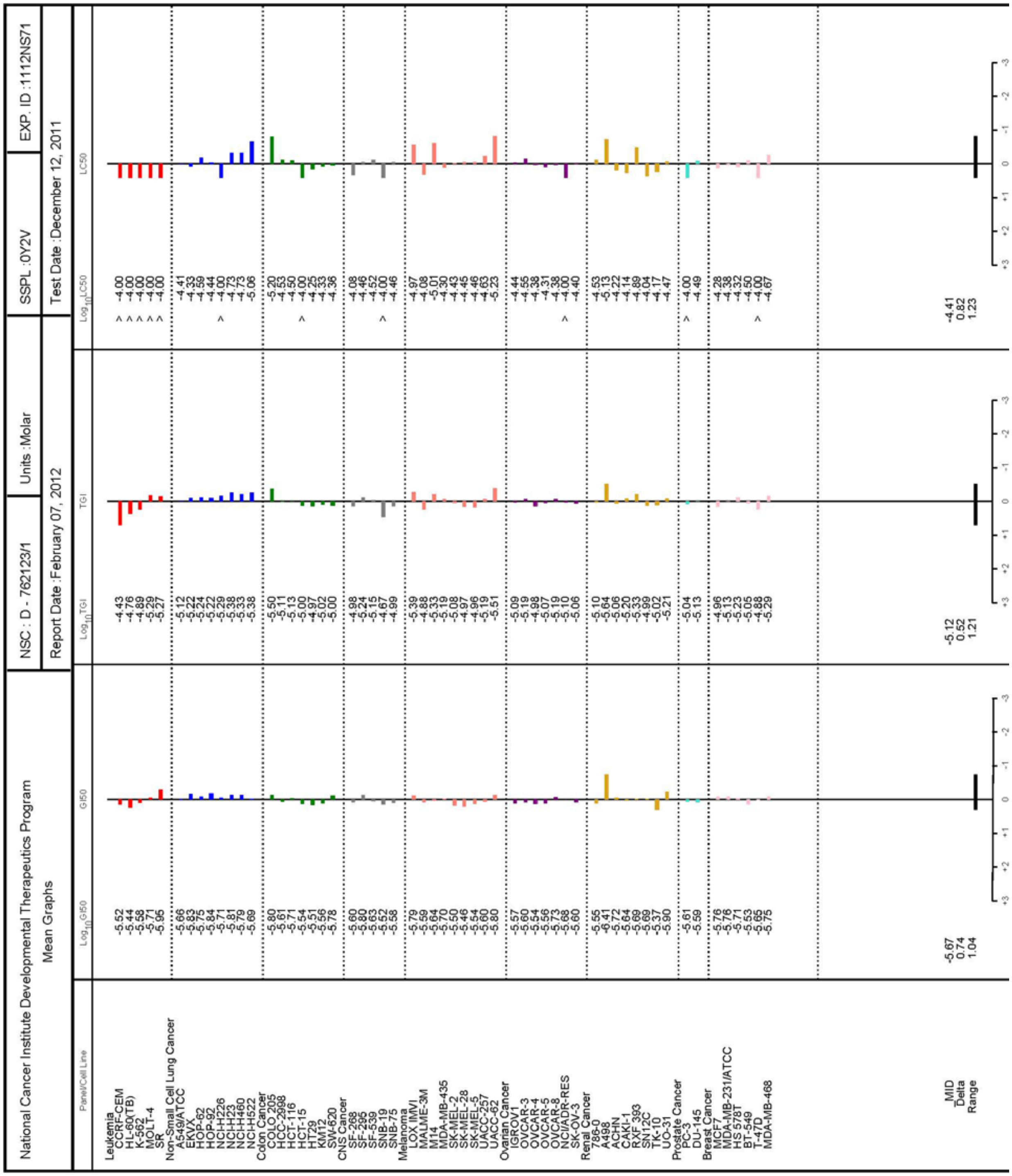

Figure S17: COMPARE Analysis data for compound 13 (Black Bars) in direct comparison with SCH1473759 (NSC761691, Red Lines), an Aurora kinase inhibitor.

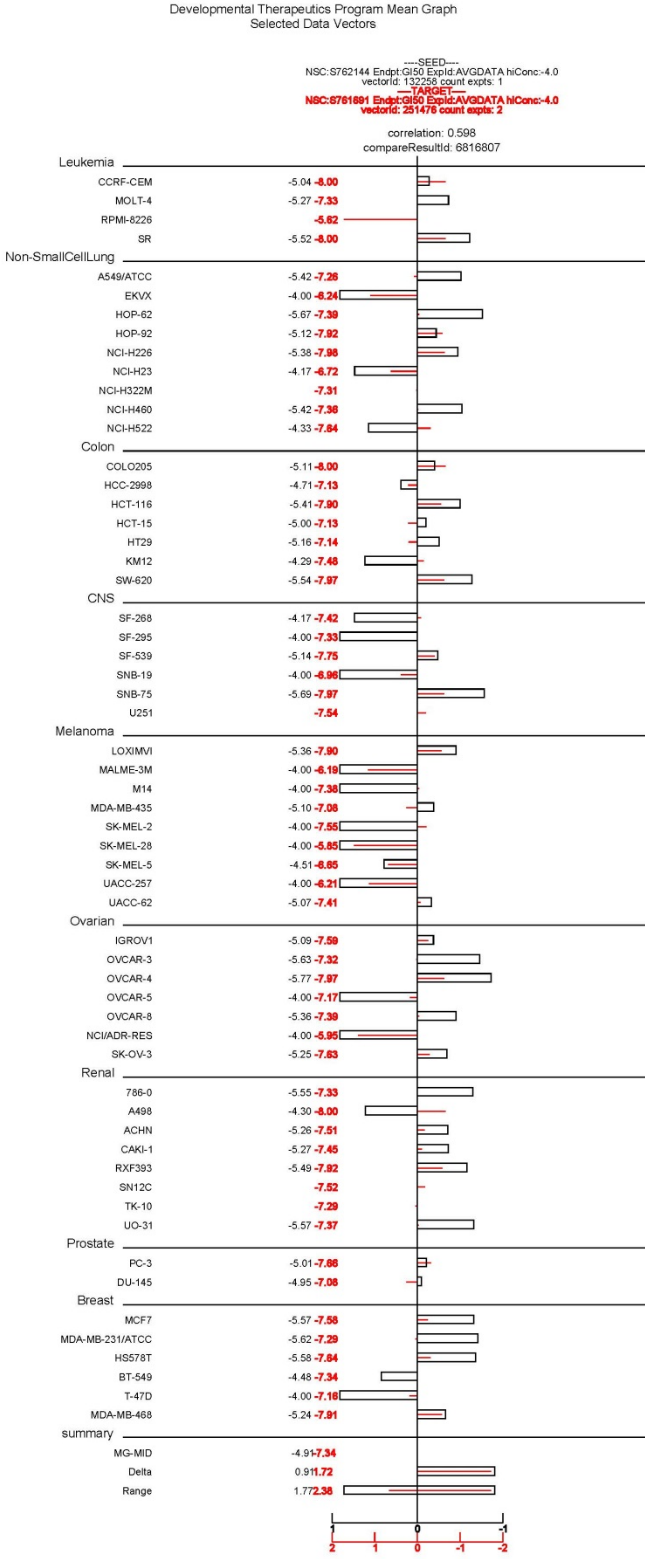

Supplement: Supplementary file 1 [file pharmaceuticals-12-00090-s001.pdf]
